# Vertebrate-tropism of a cressdnavirus lineage implicated by poxvirus gene capture

**DOI:** 10.1073/pnas.2303844120

**Published:** 2023-05-08

**Authors:** Cormac M. Kinsella, Lia van der Hoek

**Affiliations:** ^a^Amsterdam University Medical Centers (UMC), Laboratory of Experimental Virology, Department of Medical Microbiology and Infection Prevention, University of Amsterdam, 1105 AZ Amsterdam, The Netherlands; ^b^Amsterdam Institute for Infection and Immunity, 1100 DD Amsterdam, The Netherlands

**Keywords:** *Draupnirviridae*, *Krikovirus*, *Avipoxvirus*, horizontal gene transfer, vertebrate host

## Abstract

Among cressdnaviruses, only the family *Circoviridae* is recognized to infect vertebrates, while many others have unknown hosts. Detection of virus-to-host horizontal gene transfer is useful for solving such virus–host relationships. Here, we extend this utility to an unusual case of virus-to-virus horizontal transfer, showing multiple ancient captures of cressdnavirus *Rep* genes by avipoxviruses—large dsDNA pathogens of birds and other saurians. As gene transfers must have occurred during virus coinfections, saurian hosts were implied for the cressdnavirus donor lineage. Surprisingly, phylogenetic analysis revealed that donors were not members of the vertebrate-infecting *Circoviridae*, instead belonging to a previously unclassified family that we name *Draupnirviridae*. While draupnirviruses still circulate today, we show that those in the genus *Krikovirus* infected saurian vertebrates at least 114 Mya, leaving endogenous viral elements inside snake, lizard, and turtle genomes throughout the Cretaceous Period. Endogenous krikovirus elements in some insect genomes and frequent detection in mosquitoes imply that spillover to vertebrates was arthropod mediated, while ancestral draupnirviruses likely infected protists before their emergence in animals. A modern krikovirus sampled from an avipoxvirus-induced lesion shows that their interaction with poxviruses is ongoing. Captured *Rep* genes in poxvirus genomes often have inactivated catalytic motifs, yet near-total presence across the *Avipoxvirus* genus, and evidence of both expression and purifying selection on them suggests currently unknown functions.

## Significance

A single family of cressdnaviruses is known to infect vertebrates, the *Circoviridae*. Here, we identified a second that has historically infected saurians, naming them the *Draupnirviridae*. The initial clue was that some draupnirviruses donated their *Rep* gene to poxviruses exclusively infecting birds and their relatives. Since this implied the donors also infected vertebrates, a search for draupnirvirus-derived endogenous viral elements was done in ~25,000 eukaryotic genome assemblies. This confirmed that some draupnirviruses infected saurian vertebrates as long ago as the Cretaceous Period, over 100 million years before present, and they still circulate today. We propose that their evolutionary path likely began with protist-infecting ancestors, followed by emergence in insects, and eventual transfer to vertebrates by blood feeders.

The majority of newly identified viral genomes are from “stray viruses,” which we define as those with known genome sequences but with unknown hosts. This situation arose due to widespread application of metagenomic high-throughput sequencing, an efficient culture-independent virus discovery method (1, 2). When applied to samples containing diverse lifeforms, linkage of viruses to specific hosts is challenging. Identifying hosts is essential to understanding virus evolution and their medical or ecological roles (3). Viruses of eukaryotes with circular single-stranded DNA (ssDNA) genomes and a homologous replication-associated protein (Rep) are classified in the phylum *Cressdnaviricota* (4), currently containing 11 official families (5) and 46 unclassified lineages (3, 6, 7). Across the phylum, only some members of the family *Circoviridae* are recognized to infect vertebrates, most notably the pathogens porcine circovirus 2 (PCV2) and beak and feather disease virus (BFDV), infecting pigs and birds, respectively (8–10).

Fossils in eukaryotic genomes known as endogenous viral elements (EVEs) are the product of horizontal gene transfer (HGT) from viruses to host germline genomes (11), and analysis of EVE genetic relationships has helped identify hosts of some stray viruses (3, 12, 13). *Circoviridae*-derived EVEs have previously been identified in vertebrate genomes including snakes and mammals, some dating back as far as 65 to 68 million years, accounting for recent host divergence estimates (11, 14, 15). Though infrequent, analogous HGT between groups of unrelated viruses has also been observed (16–18). For example, some dsDNA herpesviruses and adenoviruses possess *Rep* genes gained from ssDNA parvoviruses (19–21), the *U94* gene of human herpesvirus 6 (HHV-6) being a well-characterized example (16, 22). Since virus-to-virus HGT requires coinfection by donor and recipient, if the host of one is known, inference of the second is theoretically possible.

Upon genome sequencing of canarypox virus (CNPV, family *Poxviridae*, genus *Avipoxvirus*), sequence similarity was observed between genes *CNPV153*, *CNPV200*, and circovirus *Reps*, suggesting HGT between ssDNA cressdnaviruses and a dsDNA avipoxvirus (23). Avipoxviruses primarily infect birds (24), though other saurians including turtles and lizards are also hosts (25, 26). If *CNPV153* and *CNPV200* truly represent HGT from cressdnaviruses, this suggests the donor viruses also infected saurians. Since discovery, the CNPV *Rep*-like genes have not been further researched. With over 1,000 *Poxviridae* genome assemblies now available, detailed comparative analysis is possible. Among the *Cressdnaviricota*, multiple thousands of genomes and a revised taxonomy have also facilitated research into their diversity (4). Here, we investigated possible HGT between viral realms, showing *Rep* genes were indeed horizontally transferred to an ancestor of extant avipoxviruses. Surprisingly, we found that donor viruses belonged to the unclassified lineage CRESSV3, which we propose be officially named as the family *Draupnirviridae*. Confirming our hypothesis, we found that draupnirviruses of the genus *Krikovirus* first infected saurian hosts at least 114 Mya.

## Results

### *Rep* Was Donated by a Cressdnavirus to an Ancestor of Extant Avipoxviruses.

**Fig. 1.**
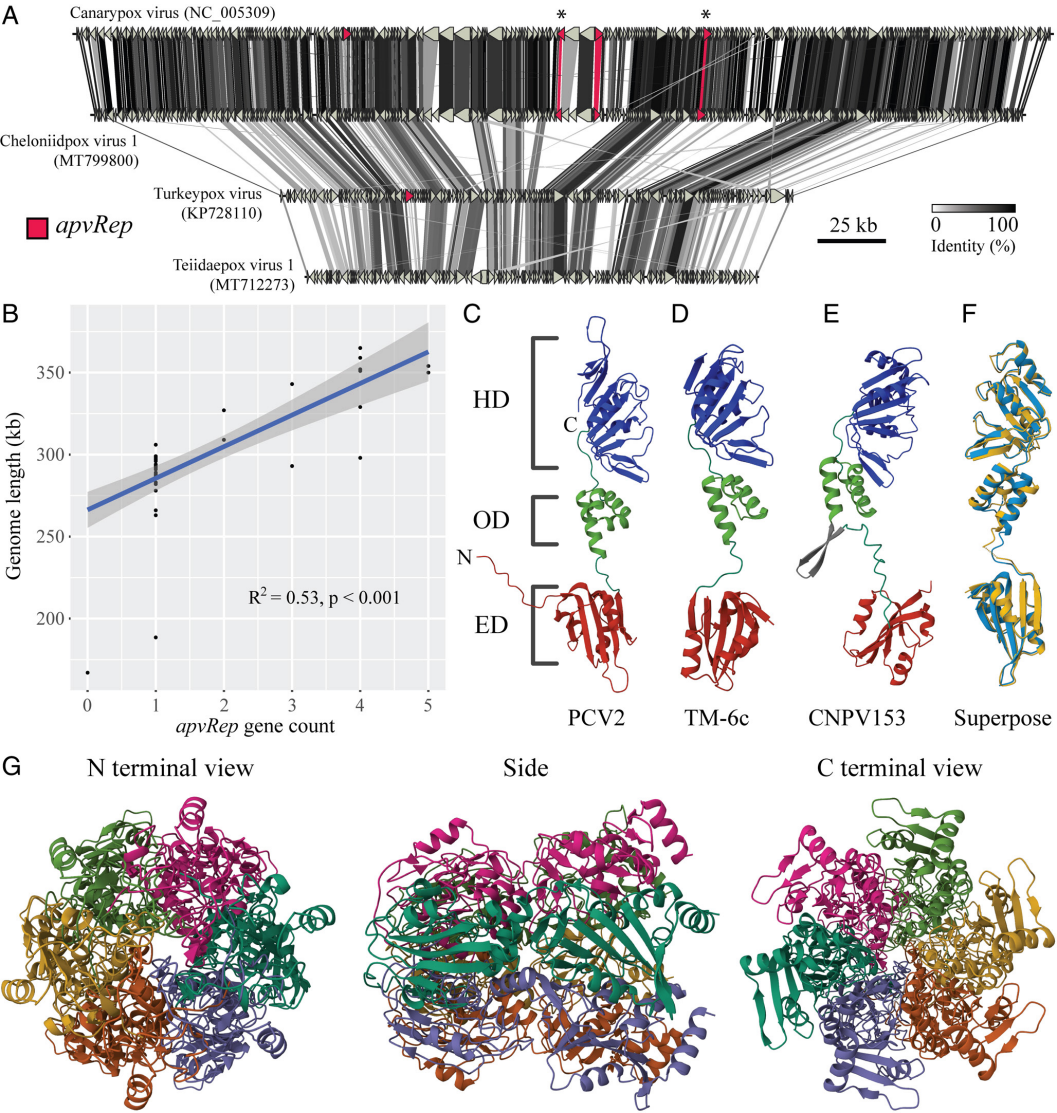
Cressdnaviruses horizontally transferred *Rep* to members of the genus *Avipoxvirus* (*Poxviridae*). (*A*) Synteny map of four avipoxvirus genomes. Red-highlighted *apvReps* have been enlarged for visibility. The two asterisked *apvReps* denote *CNPV153* and *CNPV200* (left to right respectively), discussed by Tulman et al. (23). (*B*) Scatterplot and linear regression showing the relationship between *apvRep* gene count and genome size. (*C*) AlphaFold predicted structure of the complete PCV2 Rep protein (*Circoviridae*, NP_937956.1). Domains are coloured and annotated. ED = endonuclease domain, OD = oligomerisation domain, HD = helicase domain. N and C denote the respective termini. (*D*) AlphaFold predicted structure of Rep from the prototypical krikovirus TM-6c (27, 28) (CRESSV3, ADI48253.1). (*E*) AlphaFold predicted structure of CNPV153 (*apvRep-2*, NP_955176.1). The predicted pair of antiparallel beta strands are coloured grey. (*F*) Superposed structure alignment between TM-6c Rep (cyan) and CNPV153 (gold) performed with the jFATCAT flexible algorithm; rmsd = 1.02, TM-score = 0.32, query (TM-6c) coverage 99%, target coverage 84%. (*G*) Orthogonal views of the predicted hexamer structure of CNPV153.

To detect HGT from cressdnaviruses to poxviruses, we screened 1,090 poxvirus genomes using a phylogenetically broad protein database comprising cressdnavirus Reps and Caps. We found 89 sequences with high sequence identity to cressdnavirus Reps within 51 poxvirus genomes, including that of CNPV (*SI Appendix*, Tables S1 and S2). All the 51 genomes belonged to the genus *Avipoxvirus*. Other genera were not detected, including during manual examination of *Macropopoxvirus*, the closest relative of *Avipoxvirus* (*SI Appendix*, Fig. S1*A*), and *Crocodylidpoxvirus*, the other genus infecting archosaurs (crocodiles). The 51 *Rep*-like+ genomes represent all but one sequenced avipoxviruses, as teiidaepox virus 1 (TePV-1) contained none (Fig. 1*A*). Rather than ancestral absence, this likely reflects gene loss, as TePV-1 phylogenetically nests within *Rep*-like+ avipoxviruses (*SI Appendix*, Fig. S1*B*). Our result confirms that the observations of Tulman et al. (23) represent HGT, extending this to extant avipoxviruses rather than CNPV specifically. Hereafter, we refer to HGT-derived *Rep* genes as *apvRep* genes (for avipoxvirus *Rep*).

The *apvRep* gene count varied from one to five in avipoxvirus genomes, excepting TePV-1 (*SI Appendix*, Fig. S1*B*). We observed that specific genes maintained their relative genomic positions across the genus; for example, three of the four *apvReps* in CNPV are syntenic with those in cheloniidpox virus 1 (ChePV-1, Fig. 1*A*), while the fourth in CNPV is syntenic with the lone *apvRep* in turkeypox virus HU1124/2011 (TKPV HU1124). This suggests that discrete homologous *apvReps* are found across avipoxvirus genomes. Using gene synteny analyses, we could group the 89 *apvRep* sequences into five separate genes (*apvRep-1* to *apvRep-5*). We found *apvRep-1* was distinct in having alleles across 48 of 51 *apvRep*+ genomes, covering the breadth of recognized avipoxvirus diversity. This suggests that *apvRep-1* is the oldest surviving, gained in an ancestor of the genus. Notably, *apvRep* gene count follows a phylogenetic pattern; early-branching avipoxviruses contain *apvRep-1* only, while subsequent branches gained genes stepwise over time (*SI Appendix*, Fig. S1*B*). Increasing genome size may have driven or facilitated this, as early-branching avipoxviruses have short genomes (e.g., TKPV HU1124 and TePV-1, 189 and 167 kb, respectively) compared with late-branching species (e.g., CNPV and ChePV-1, 365 and 343 kb, respectively). Small genome size is likely an ancestral trait of avipoxviruses, given the genome lengths of closely related macropopoxviruses (167 to 170 kb). As predicted, *apvRep* gene count positively correlates with genome length (Fig. 1*B*), suggesting larger genomes can house and benefit from increased gene dosage.

Large variability was found in the predicted protein lengths encoded by different *apvRep* genes, for example apvRep-1 sequences ranged from 100 to 111 amino acids (aa), while some apvRep-3 sequences reached 1,006 aa (*SI Appendix*, Table S2). Sequences of apvRep-2 were predicted at lengths comparable to exogenous cressdnavirus Reps, 310 to 312 aa long. One of these was encoded by *CNPV153*, originally noted as a cressdnavirus-like gene by Tulman et al. (23). To explore structural conservation of these full-length apvReps in comparison to cressdnavirus proteins, we predicted the Rep structures of PCV2 (*Circoviridae*), TM-6c (27) (CRESSV3), and CNPV153 using AlphaFold. The PCV2 predicted structure was highly consistent with experimental solutions of all the three expected domains, the endonuclease (29, 30), oligomerization, and helicase domains (31) (Fig. 1*C*). The TM-6c prediction resembled that of PCV2 (Fig. 1*D*), though no experimental solutions from CRESSV3 are published. Overall, the predicted structure of CNPV153 matched the cressdnavirus Reps; for example, the endonuclease domains all shared a five-stranded beta sheet with two alpha helices on one side and a third on the other (Fig. 1*E*). Relaxed structure alignment between TM-6c and CNPV153 revealed broad concordance (Fig. 1*F*). However, AlphaFold predicted a pair of antiparallel beta strands 14 residues long, just N-terminal of the CNPV153 oligomerization domain, which were not seen in the cressdnaviruses (Fig. 1*E*). This prevented full TM-6c and CNPV153 alignment coverage using a rigid structure method (*SI Appendix*, Fig. S1*C*). Whether this represents prediction error or true biology is unclear, though if the latter––any disruptive effect on oligomerization would be notable. To explore this, we predicted the structure of hexameric CNPV153, the subunit count found in PCV2 Rep complexes (31). AlphaFold predicted hexameric CNPV153 to form a torus (Fig. 1*G*) with similarity to the solved PCV2 hexamer (which covers the oligomerization and helicase domains) (31). Unlike with monomer prediction, the antiparallel beta strands adjacent to the oligomerization domain were not observed, suggesting that oligomerization of CNPV153 is possible.

After finding that apvRep-2 structure is broadly conserved with cressdnavirus Reps, we investigated why other *apvRep* genes displayed high variability in predicted protein length. Most extreme among these were intact *apvRep-3* alleles, which had a bimodal length distribution, encoding either 176 to 179 aa or 827 to 1,006 aa, far longer than any canonical cressdnavirus Rep. Using a comparative genomics approach, we found that long alleles came about by gene fusion, subsequently inherited by four species (*SI Appendix*, Table S2). Illustrating this, the long allele in finchpox virus shares synteny and sequence identity with three separate open-reading frames in ChePV-1, the last of which is a short *apvRep-3* allele (*SI Appendix*, Fig. S1*D*). We did not observe a genome with only two fused genes, and thus cannot determine whether fusion occurred in one step or two. The functions of the two genes sometimes fused to *apvRep-3* are unknown. Searches for conserved domains in the unfused homologs of ChePV-1 revealed none for the first (*ChPV157*, QRI42875.1), though the second (*ChPV158*, QRI42876.1) contained domain similarity to accession cl31759 (ring-infected erythrocyte surface antigen domain, known from *Plasmodium falciparum*). An equivalent search in the fused allele of CNPV (*CNPV156*, NP_955179.1) found the same, plus a hit to cl38662 (domain of unknown function found in the roundworm class *Chromadorea*). A search of the finchpox virus-fused allele (UOX38671.1) additionally detected similarity to cl27103 (secretion system effector C-like domain, found in some bacterial pathogens). Though the biological significance of these findings is uncertain, it is notable that *CNPV156* is found in a virulence and host range–related genomic region (23).

**Fig. 2.**
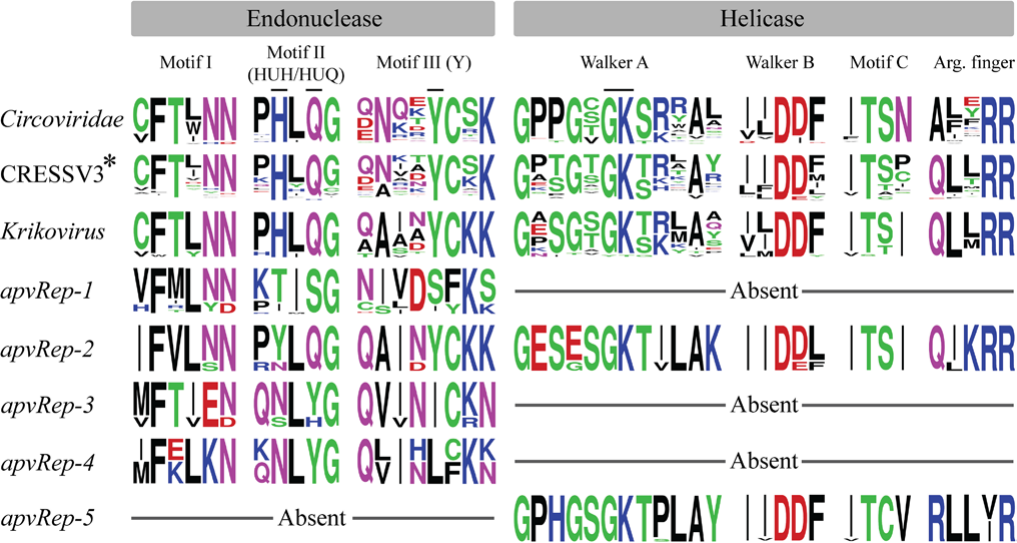
Rep protein sequence motifs in cressdnaviruses and avipoxviruses. Asterisked lineage CRESSV3 (also referred to as *Draupnirviridae* in this study) includes members except for *Krikovirus*, which is shown separately. Arg. = arginine. Residue colours: hydrophobic = black, polar = green, basic = blue, acidic = red, neutral = purple. Key residues discussed in the main text are marked.

After accounting for the high length of some alleles, we investigated those shorter than typical Rep proteins. Seven of nine collective *apvRep* alleles from crowpox virus, magpiepox virus 1, and magpiepox virus 2 (ON408417.1, MK903864.1, and MW485973.1) were either fragmented by stop codons, truncated relative to homologous alleles in other species, or missed start codons, evidence of pseudogenization. The three genomes are closely related (*SI Appendix*, Fig. S1*B*), suggesting that a lineage-specific loss of several *apvRep* genes is ongoing. Similar sequence degradation was not observed in *apvReps* of other species, though we noted that apart from the full-length *apvRep-2* already discussed, all genes were simplified in terms of their domain architecture. They encoded either the endonuclease (*apvRep-1, apvRep-3,* and *apvRep-4*) or the helicase (*apvRep-5*) (Fig. 2 and *SI Appendix*, Fig. S2), explaining alleles with low predicted length. The finding implies the five *apvRep* genes originated from at least two gene capture events, as *apvRep-1* lost its helicase domain in an ancestor of all extant avipoxviruses, and thus is not a paralog of either *apvRep-2* or *apvRep-5*, which appeared later in avipoxvirus evolution and possess a helicase. The majority of *apvRep* alleles lack the oligomerization domain (*SI Appendix*, Fig. S2), suggesting normal Rep functionality is altered or absent. Cressdnavirus Rep proteins require nuclear import for normal replicative functions, and for PCV2 Rep, the nuclear localization signal is within the 20 N-terminal residues (32). The equivalent residues are absent or contain a sizable deletion in all *apvRep* alleles, consistent with the cytoplasmic localization of poxviruses. Furthermore, some key functional motifs of Rep proteins are not conserved in apvRep sequences. The endonuclease active site HUH motif crucial for ssDNA cleavage activity (HUQ in *Circoviridae* (33) and several other cressdnavirus lineages) is inactivated in all *apvRep* genes with endonuclease domains, while the key tyrosine residue that covalently binds the 5′ phosphate of cleaved ssDNA is missing in all but *apvRep-2* (Fig. 2) (34). The helicase motifs of *apvRep-2* and *apvRep-5* appear more conserved overall, including the key GK residues used in nucleotide binding of the Walker A motif, and Walker B residues involved in ATPase activity (35). However, arginine finger sequences were not fully conserved, with potential impacts on nucleotide hydrolysis. Overall, full canonical enzymatic activity by apvRep proteins appears unlikely, though the potential for some nucleotide interaction may remain. These findings are remarkably similar to the U94 protein of HHV-6, which has inactivated endonuclease motifs but a conserved Walker A and B, and partial conservation of other helicase motifs (*SI Appendix*, Fig. S3), compatible with experimental results showing endonuclease inactivity yet retention of several helicase functions (22).

**Fig. 3.**
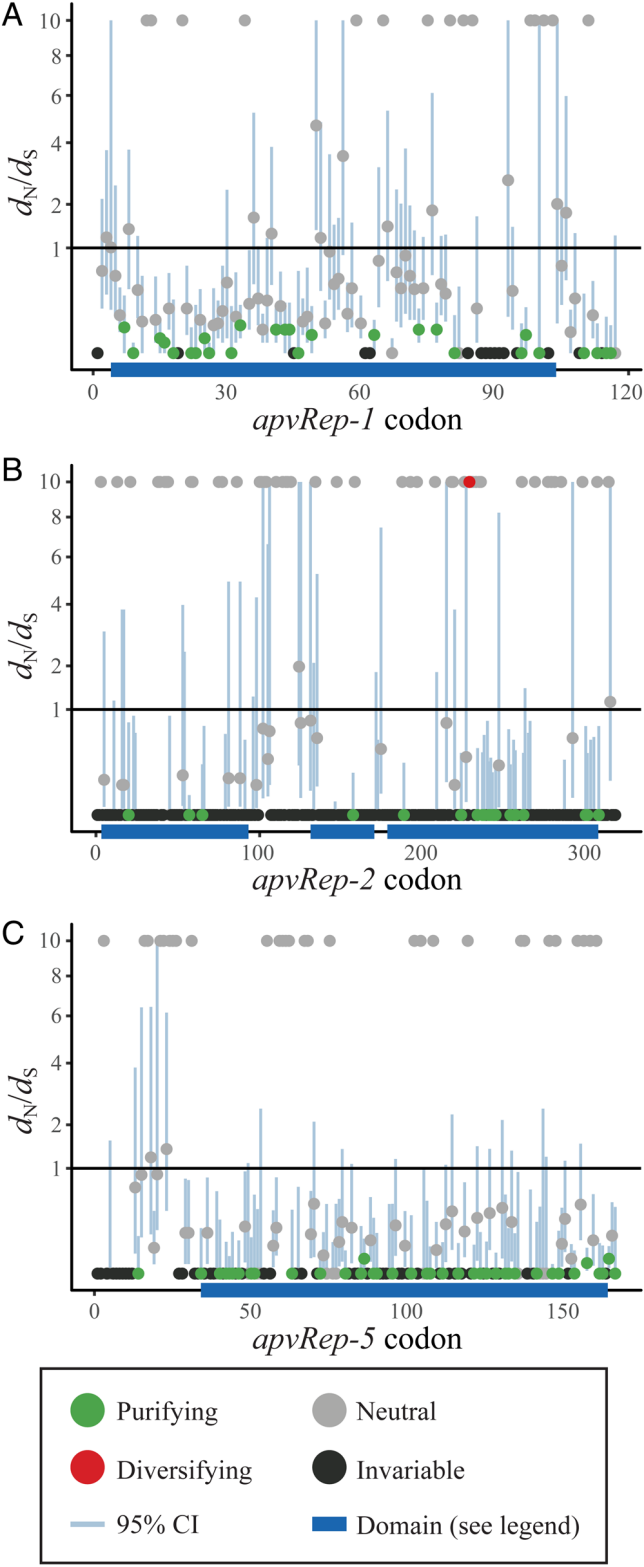
Purifying selection has acted on *apvReps*. (*A*) Site-by-site *d*_N_/*d*_S_ estimates for *apvRep-1*. The endonuclease domain is annotated. Statistically significant difference from null (neutrality, i.e., *d*_N_/*d*_S_ = 1) required *P* ≤ 0.05. (*B*) Site-by-site *d*_N_/*d*_S_ estimates for *apvRep-2*. From left to right, the endonuclease, oligomerisation, and helicase domains are annotated. (*C*) Site-by-site *d*_N_/*d*_S_ estimates for *apvRep-5*. The helicase domain is annotated.

That apvRep sequences vary dramatically in their represented domains and often have canonical functional motifs inactivated may appear contradictory with their near-total presence across the genus *Avipoxvirus*, and conservation since the common ancestor, which together suggest *apvRep* presence enhances viral fitness. To examine whether they could be pseudogenes (except those seven already discussed), we looked for evidence of their expression. Using published RNA sequencing data (PRJNA524335) from cell cultures infected for 16 h with either CNPV or fowlpox virus (FPV) (36), we confirmed that at least *apvRep-2*, *apvRep-3*, and *apvRep-5* are expressed by CNPV, while *apvRep-1* was silent (*SI Appendix*, Fig. S4). Interestingly, *apvRep-1* was expressed in FPV, which lacks other *apvReps*, and this difference may be due to the genetic redundancy found in CNPV. While no proteomic data was available to analyze *apvRep* translation, we found indirect evidence via purifying selection on *apvRep* coding sequences—including those encoding only the endonuclease (*apvRep-1*), all three domains (*apvRep-2*), or only the helicase (*apvRep-5*). Global *d*_N_/*d*_S_ ratios were estimated at 0.23, 0.33, and 0.17, respectively, indicating purifying selection has acted to conserve each coding sequence and implying translation occurs. At the site level, the majority of *apvRep-1* codons had an estimated *d*_N_/*d*_S_ < 1 and many were statistically significant for purifying selection (Fig. 3*A*). These tended to cluster on the endonuclease domain itself, and also the codons C-terminal of it. We noted that *apvRep-1* also had comparatively few invariant sites (Fig. 3 *A–C*), though alleles were available from most avipoxvirus species, and this likely introduced more variation. Both factors probably reflect its relatively old age. Sites of *apvRep-2* were mostly invariant (Fig. 3*B*), though it is found in relatively few species and is the only fully intact *apvRep*, suggesting evolutionary youth. Despite this, we still observed evidence of purifying selection on some sites across the gene. Notably, the first residue of what was once the endonuclease HUH/HUQ motif may have experienced diversifying selection, inactivating it; however, the *p-*value did not reach significance (0.059). One residue just C-terminal of the Walker B motif also reached significance for diversifying selection, though the possible impact on helicase activity is unclear. The *apvRep-5* gene displayed strong evidence of purifying selection targeting the helicase domain, which was dense with sites significantly below *d*_N_/*d*_S_ of 1 (Fig. 3*C*). Overall, the evidence suggests *apvReps* of each domain architecture experience purifying selection on the peptide sequence and are not pseudogenes. Given the tendency for selection to target the functional domains themselves, it appears likely that tertiary structure is being maintained. With evidence suggesting canonical enzymatic Rep functionality is disrupted, *apvRep* genes may instead provide nonenzymatic Rep functions or have undergone exaptation.

### Cressdnavirus *Rep* Donors Belong to a New Cressdnavirus Family, *Draupnirviridae*.

**Fig. 4.**
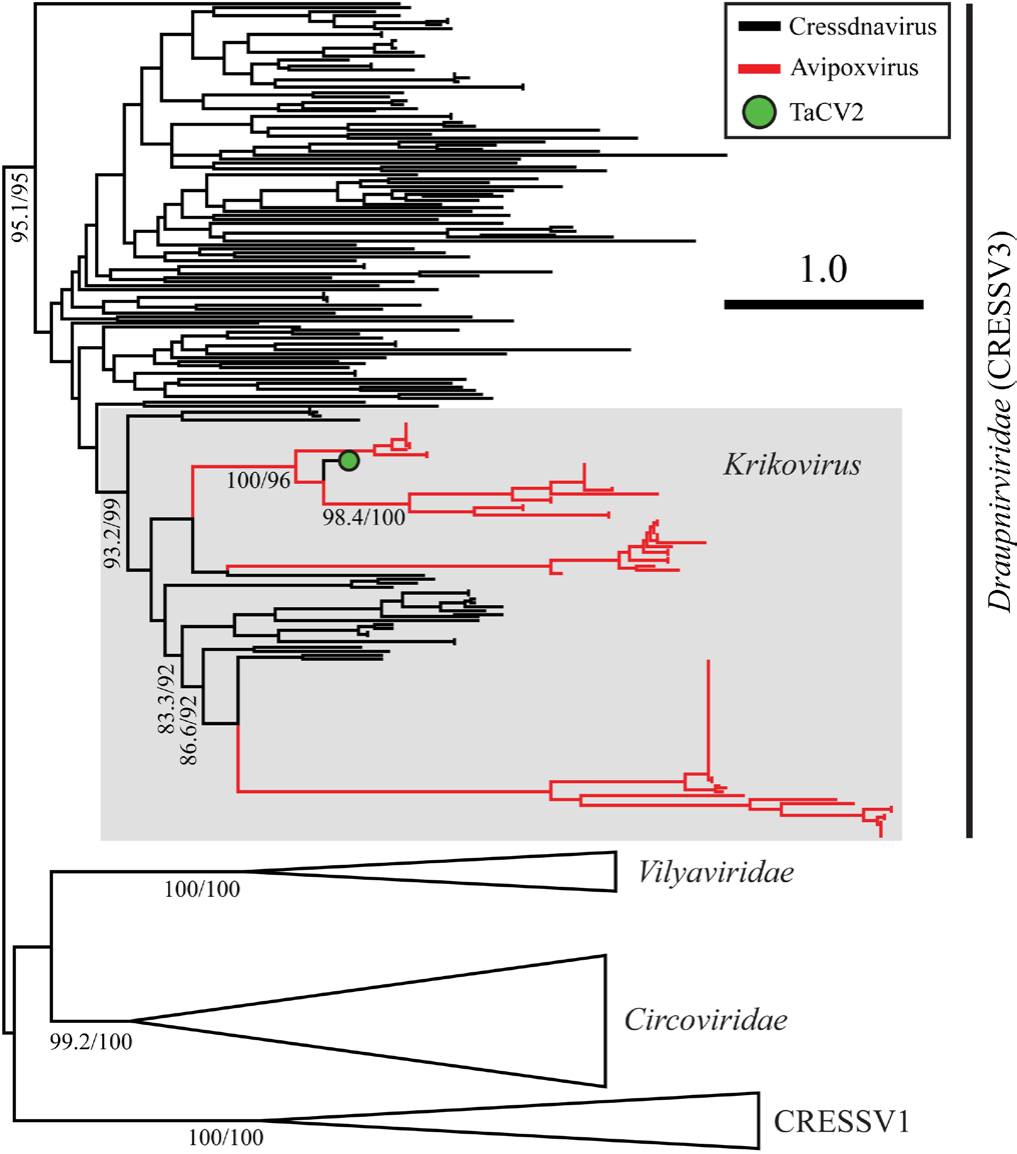
Maximum-likelihood phylogeny of selected Rep lineages. All apvRep sequences belong to the genus *Krikovirus* (grey box), within the *Draupnirviridae* (i.e., CRESSV3). TaCV2 = Tanager-associated CRESS DNA virus 2 (MF804498.1). Scale bar is in amino acid substitutions per site. Branch supports report Shimodaira Hasegawa approximate likelihood-ratio test (SH-aLRT) scores on the left and ultrafast bootstrap scores on the right.

Since avipoxviruses infect saurian hosts and cressdnaviruses have donated *Reps* to them, we predicted the donor lineage would also infect saurians. We thus expected them to fall within the vertebrate-infecting *Circoviridae*, yet phylogenetic analysis alongside all cressdnavirus lineages resolved apvRep sequences within the unclassified lineage CRESSV3 (7) (*SI Appendix*, Fig. S5). A second more focused phylogeny confirmed that apvRep proteins belong to CRESSV3, specifically within the previously proposed genus *Krikovirus* (28) (Fig. 4). Our analyses thus robustly support *Krikovirus* as a monophyletic lineage within CRESSV3 (Fig. 4 and *SI Appendix*, Fig. S6), and as the lineage that donated *Reps* to avipoxviruses.

In line with the step-wise gain of *apvRep* genes already discussed, phylogenetic analysis hinted at multiple independent captures of *Rep* genes by avipoxviruses (Fig. 4). Notably, two Reps from exogenous krikoviruses appeared more closely related to some apvReps than to other krikovirus Reps, suggesting proximity to original donor viruses. These came from Tanager-associated CRESS DNA virus 2 (TaCV2, MF804498.1) and bat circovirus isolate BtPa-CV-3/NX2013 (KJ641729.1). Strikingly, TaCV2 was sampled directly from an avipoxvirus-induced cutaneous lesion on the foot of a bird, *Thraupis episcopus* (37), showing that an interaction between avipoxviruses and krikoviruses is ongoing. The isolate BtPa-CV-3/NX2013 was sequenced from an insectivorous bat, probably from stool. Across the *Krikovirus* genus, a distinct isolation source pattern was apparent; of 28 available genomes, 14 were bat associated (stool or undescribed sample type), six were bird associated (stool and the avipoxvirus-induced lesion), and the remaining eight were insect associated, six from mosquitoes (*SI Appendix*, Table S3). This contrasted with other members of CRESSV3, 76% of which were associated with water or aquatic life. We hypothesize that bat stool-associated krikoviruses represent ingested insect-associated viruses. Krikoviruses in bird stool may also have been ingested, or instead were shed virus. Based on evidence that krikoviruses have an ancient yet ongoing relationship with avipoxviruses, we hypothesized that they share saurian hosts, since HGT would require coinfection of the same host. Alternatively, mosquitoes may represent another setting where HGT could have occurred, as they carry both krikoviruses and avipoxviruses (28, 38, 39).

In light of our phylogenetic analyses and investigations into the hosts of both krikoviruses and the wider CRESSV3 lineage (see below), we propose that a cressdnavirus family be created to replace the temporary name CRESSV3. We suggest the name *Draupnirviridae*. The name comes from the ring Draupnir of Norse mythology, said to have multiplied itself every ninth night. It alludes to both the circular viral genome and genome replication. Furthermore, we support the proposal of Garigliany et al. (28), insofar as *Krikovirus* should be an official cressdnavirus genus, and we propose this should be within the *Draupnirviridae*. In this report, we use the name *Draupnirviridae* instead of CRESSV3 hereafter.

### Draupnirviruses Have Infected Saurians for Millions of Years.

**Fig. 5.**
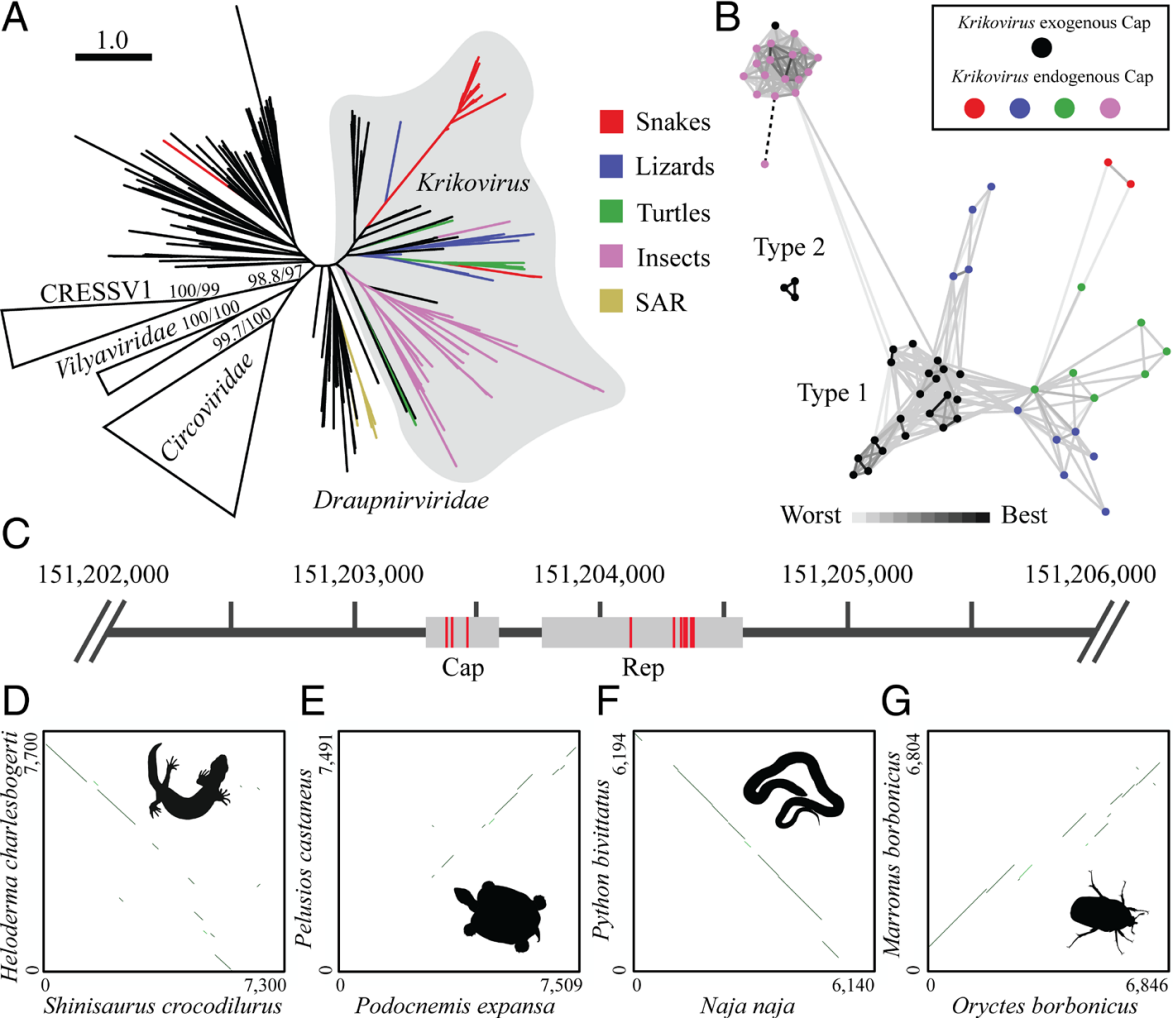
Saurian genomes contain ancient endogenous krikovirus-derived elements. (*A*) Maximum-likelihood phylogeny of selected Rep protein lineages within *Cressdnaviricota*. CRESSV1, *Vilyaviridae*, and *Circoviridae* serve as outgroups for the *Draupnirviridae.* EVEs extracted from eukaryotic genome assemblies are shown as coloured branches. SAR refers to stramenopiles, alveolates, and rhizarians. Scale bar is in amino acid substitutions per site. Branch supports report SH-aLRT scores on the left and ultrafast bootstrap scores on the right. (*B*) Clustered krikovirus Caps including exogenous and endogenous sequences. Connections represent BLASTp alignments, with shade denoting significance level (maximum/worst e-value = 1e^−10^). The dotted line connects a Cap to known relatives despite no significant BLASTp alignment using CLuster ANalysis of Sequences (CLANS). (*C*) Integration of a complete krikovirus genome into chromosome 2 of *S. crocodilurus* (CM037877.1). Regions with alignment to query krikovirus proteins are shown in grey. Red bars indicate stop codons. (*D*) LAST alignment dotplot of shared krikovirus EVE and sequence context in *S. crocodilurus* (151,200,291-151,207,590 in CM037877.1) and *H. charlesbogerti* (1,842,167-1,849,871 in JANEZZ010002294.1). EVE sequence was masked prior to alignment. (*E*) Shared krikovirus EVE and sequence context in *P. expansa* (26,222,225-26,229,734 in ML681998.1) and *P. castaneus* (5,808,247-5,815,738 in ML685784.1). (*F*) Shared krikovirus EVE and sequence context in *N. naja* (297,176,533-297,182,673 in CM019148.1) and *P. bivittatus* (36,161-42,355 in NW_006537177.1). (*G*) Shared krikovirus EVE and sequence context in *O. borbonicus* (2,594-9,440 in LJIG01000918.1) and *M. borbonicus* (18,407,007-18,413,811 in LR737382.1). Animal silhouettes were retrieved from phylopic.org (*Heloderma* representing *Anguimorpha* by Nicolas Mongiardino Koch, *Stupendemys* representing *Pleurodira* by Roberto Díaz Sibaja, *Rhabdophis* (i.e., *Macropisthodon*) representing *Serpentes* by V. Deepak, and *Cotinis* representing *Dynastinae* by C. Camilo Julián-Caballero). Silhouettes were available in the public domain or under a creative commons license (https://creativecommons.org/licenses/by/3.0).

We hypothesized that krikoviruses (family *Draupnirviridae*) infect saurian hosts, based on evidence that they donated *Reps* to saurian-infecting avipoxviruses. To explore this, we carried out a detailed search for draupnirvirus-derived EVEs in nearly all available eukaryotic genome assemblies. After quality curation, a total of 145 *Rep*-like and 38 *Cap*-like EVEs were identified, often in long scaffolds or chromosome-resolved assemblies. In line with expectations for EVE sequences (12), the guanine-cytosine (GC) contents of endogenous elements were lower than those of homologous exogenous viruses (*SI Appendix*, Fig. S7). Of the *Rep-*like sequences, 133 belonged to *Krikovirus*, 11 to *Draupnirviridae* (but were not assignable to *Krikovirus*), and the last was discarded due to inconsistent phylogenetic placement (Fig. 5*A*). The krikovirus-like sequences were found in the genome assemblies of 47 species, all either saurians or insects. These included 37 snakes, 3 lizards, 2 turtles, 4 beetles, and 1 earwig (*SI Appendix*, Table S4). Together, they suggest a saurian and insect host range for krikoviruses, in line with both theoretical prediction and observed isolation sources. The 11 draupnirvirus-like EVE sequences outside of *Krikovirus* were resolved in two sections of the tree. Two short (≤48 aa) sequences came from the snake *Anilios bituberculatus*, and may be phylogenetically misplaced krikoviruses or even circoviruses. The other nine sequences came from *Chromera velia* and *Polymyxa betae*, both of the stramenopiles, alveolates, and rhizarians (SAR) supergroup of protists. This suggests that draupnirviruses outside *Krikovirus* infect various protists, with the ancestral krikoviruses spilling over into animals. Cap proteins of sequenced exogenous krikoviruses belong to two lineages; the lineage found in the majority of genomes is *Circoviridae*-like based on HHpred analysis (40) (type 1, e.g., ARO38299.1), while the other has ambiguous ancestry and is found in three genomes (type 2, e.g., QKN88852.1). Of the 38 *Cap*-like EVEs identified, 36 were related to type 1 krikovirus Caps and were found in genomes of snakes, lizards, turtles, and insects (Fig. 5*B*). None were related to type 2 Caps. While related, *Cap*-like EVEs in insect and vertebrates separated during cluster analysis, suggesting some Cap-mediated host tropism may occur, or that there were phylogenetic biases in the progenitor viruses depositing EVEs in respective host lineages. The last two *Cap*-like EVEs belonged to lineages sometimes found with draupnirvirus Reps and were again found in *C. velia* and *P. betae. Krikovirus Cap*-like EVEs were often found paired with *Krikovirus Rep*-like EVEs (17 out of 36 cases), showing they were integrated as whole virus genomes (Fig. 5*C*). EVEs of both gene types regularly contained numerous stop codons, suggestive of ancient origin; for example, one integration in *Shinisaurus crocodilurus* chromosome 2 had three stops in the predicted *Cap* sequence and seven in the *Rep* (Fig. 5*C*).

The indication of ancient krikovirus-derived EVEs led us to explore their possible homology between host species. After masking individual EVE sequences, we carried out all-versus-all alignment of sequence contexts around each integration site, finding many were homologous to each other (*SI Appendix*, Table S4). Time-calibrated host phylogenies allowed some to be temporally constrained. We found that an EVE in the neoanguimorph lizard *Heloderma charlesbogerti* is shared with the paleoanguimorph *S. crocodilurus* (Fig. 5*D*), indicating that integration occurred prior to species divergence during the Cretaceous, ~114 Mya (41). A separate integration observed in the two pleurodiran turtles *Pelusios castaneus* (*Pelomedusidae*) and *Podocnemis expansa* (*Podocnemididae*) (Fig. 5*E*) predated species divergence ~112.5 Mya (42). An integration found shared between many snake species including *Python bivittatus* and *Naja naja* (Fig. 5*F*) can be dated to at least ~65 Mya, before the rapid expansion in snake diversity precipitated by the Cretaceous-Tertiary mass extinction (15, 43). While no time-calibrated phylogeny was available for the *Dynastinae* subfamily of beetles within the *Scarabaeidae*, we did observe homologous integrations, for example between *Marronus borbonicus* and *Oryctes borbonicus* (Fig. 5*G*).

## Discussion

Of 57 named cressdnavirus lineages, 14 have known or proposed hosts for some representatives (3, 44), while the rest contain stray viruses with unknown hosts. One of these is the *Circoviridae*, members of which cause severe disease in some vertebrates. Here, we presented the likely hosts of a 15th lineage, which we named as the family *Draupnirviridae*, only the second recognized to have infected vertebrates. Among draupnirviruses, the genus *Krikovirus* is linked by HGT to saurian vertebrates, insects, and strikingly avipoxviruses. Modern infection of saurians remains to be directly confirmed, though identification of TaCV2 in a bird lesion could represent viral shedding during a coinfection with an avipoxvirus (37). The remainder of the *Draupnirviridae* remains poorly characterized, though some detected EVEs suggest protistan hosts. A possible scenario for krikovirus evolution is emergence of protistan viruses into animals such as insects, followed by spillover into vertebrates. We show krikoviruses infected saurians over 100 Mya; therefore, any such spillover was ancient. Hematophagous mosquitoes are at least 100 million years old (45, 46) and can precipitate spillover by vectoring viruses (47). The possibility that mosquitoes spread krikoviruses to vertebrates is concordant with their occurrence in modern mosquitoes, as well as the donation of krikovirus *Reps* to avipoxviruses, also vectored by mosquitoes (38, 39). However, it remains uncertain whether HGT occurred in an insect or a vertebrate host, and mosquito detection may also be derived from bloodmeals. Future work should test vector competency of mosquitoes for krikoviruses and establish whether genome replication occurs in their midgut and salivary glands.

We observed krikovirus-derived *apvReps* across the genus *Avipoxvirus*, large dsDNA pathogens of conservation and animal welfare concern. At least one *apvRep* is found in all sequenced members of the genus except TePV-1, which has likely experienced gene loss. Three additional species closely related to each other are currently undergoing pseudogenization of most of their *apvRep* alleles, and together this shows gene loss is nonlethal. However, near-total *apvRep* presence across avipoxviruses, evidence of RNA expression, and purifying selection on coding sequences all suggest they are active and generally enhance viral fitness. The observed temporal expansion in *apvRep* gene count alongside genome size is also notable, and it is possible they have contributed to poxvirus adaptive evolution (48). Four of five *apvRep* genes have a simplified domain architecture, a process often seen in horizontally acquired virus genes, hypothesized to mimic or interfere with canonical functions of intact homologs (49). Given that different domains are represented, exact functions are difficult to interpret and likely vary by gene. While not directly comparable, the *Rep* gene *U94* gained by HHV-6 from a parvovirus has diverse co-opted functions, for example in viral latency (50, 51). We observed that in both *apvReps* and *U94*, sequence motifs involved in endonuclease activity are inactivated, while helicase motifs are partly conserved. Instead of canonical cressdnavirus Rep functions in rolling circle replication, it is possible that apvReps have undergone exaptation, or retain nonenzymatic Rep functions. If krikovirus Reps are inhibitory to avipoxvirus replication in the same way the homologous (33) Reps of adeno-associated viruses are to both adenoviruses and herpesviruses (52, 53), then this function may have been co-opted by avipoxviruses for regulation of genome replication, perhaps explaining higher *apvRep* count in longer genomes. Alternatively, apvReps may serve in antiviral defense against coinfecting krikoviruses. If they can disrupt krikovirus Rep complexes via a dominant negative effect, then avipoxviruses could limit krikovirus genome replication and protein production, which might inhibit their own. Devaluing this hypothesis, we found apvRep proteins are likely localized to the cytoplasm, while cressdnavirus Reps localize to the nucleus (32, 54). Krikovirus-derived EVEs in animal genomes show nuclear replication has occurred historically, as would be expected. Our study complements examples of *Rep* capture by dsDNA viruses, yet no study has shown similar evidence for *Cap* transfer. Rather than a mechanistic bias, we suspect this reflects survivorship bias—in that *Rep* is more often advantageous to recipients, and thus maintained during evolution. Given the different localization of genome replication for cressdnaviruses and poxviruses, we suspect HGT is mediated by retrotransposition of single-gene transcripts, recently confirmed to occur in poxviruses (55, 56).

Using the known host range of unrelated viruses, we used interrealm HGT to infer hosts of some stray viruses. We presume such long distance HGT is uncommon; however, we have not exhaustively characterized virus-to-virus HGT events, and thus additional virus–host relationships may be solved by doing so. While we cannot infer whether the first *apvRep* originated in a saurian host or a mosquito, it was apparently gained by an ancestor of all avipoxviruses circulating today. Estimates of ancient divergence times for viral lineages are prone to underestimation due to substitution saturation effects over long timeframes (57, 58), and high levels of genetic saturation have been observed in core poxvirus genes (59). Published estimates of divergence times between CNPV and FPV fall within the last 100 thousand years (60, 61); however, these estimates are liable to increase upon correction for substitution saturation and inclusion of early-branching avipoxvirus genomes such as TePV-1 and TKPV HU1124 (26, 62). Even so, given that krikoviruses first infected saurians over 100 Mya, their interaction with avipoxviruses appears comparatively young. Further research on this system is warranted to determine any functions of *apvReps*, the nature of krikovirus-avipoxvirus relationships, and the pathogenic potential of krikoviruses.

## Materials and Methods

### Detection of HGT to the *Poxviridae*.

A computational HGT detection workflow was designed, available from: https://github.com/CormacKinsella/HGT_finder (63). It requires a protein query; we used a phylogenetically broad cressdnavirus database of 2,923 Rep and 2,122 Cap sequences. The other input is a list of genome assemblies to process; ours contained 1,090 poxvirus assemblies available in July 2022 from GenBank and RefSeq databases. The workflow iteratively processes assemblies, handling assembly download, corruption testing, and replacement if necessary. Features aligning to query proteins are identified with tBLASTn (64) with e-value set to 1e^−5^, and alignment coordinates are converted to BED format with ascending ranges. Strictly overlapping alignments are merged with BEDTools (65) to generate a minimum–maximum coordinate range for each feature, which is extracted as a nucleotide FASTA. For each feature, the predicted protein sequence is recorded using the single best tBLASTn alignment, which can align past frameshifting mutations and retains stop codons as asterisks. For analysis of proximal or tandem features, a further BED file is generated merging features ≤1 kb apart, while for analysis of feature context, a BED and corresponding FASTA is created allowing ≤1 kb between features and appending 3 kb of sequence context to each end, scaffold length permitting. Finally, the workflow removes unrequired files and proceeds to the next assembly. Potential HGT-derived features were aligned to the GenBank nr database using DIAMOND BLASTp v2.0.15 (66) set to “--ultra-sensitive --max-target-seqs 50” to ensure reciprocal cressdnavirus alignment. Manual curation ensured all avipoxvirus *apvRep* elements were detected and complete, utilizing the NCBI tBLASTn tool and GenBank assembly annotations.

### Comparative Genomics and Characterization of *apvRep* Genes.

Comparative genomic analyses used clinker (67). Protein structures were predicted using AlphaFold v2.1.1 (68), aligned using the Protein Data Bank (PDB) pairwise structure alignment tool (69), and visualized using Mol* (70). Presence of apvRep functional domains was assessed at the structural level using AlphaFold predictions, and visually in Jalview (71) after alignment of sequences to the Reps of BFDV (ADN80874.1) and TaCV2 (AVH76405.1). For assessment of sequence motif conservation, sequence logos were generated using WebLogo (72). Possible domains in gene fusion partners were assessed using CD-Search (73). For phylogenetic analyses, regions of apvRep proteins gained by gene fusion were manually trimmed prior to alignment with cressdnavirus references using MAFFT v7.487 (74), and analysis with IQ-TREE v2.2.0 (75). Sequence GC contents were calculated using the geecee tool within EMBOSS v6.6.0.0 (76). To test for selection, *apvRep* alleles per gene were first deduplicated at the species level, degraded sequences were discarded, and sequences were aligned using MAFFT as above. Alignments were analyzed using the fixed-effects likelihood method (77), allowing synonymous rate variation and performing bootstrap resampling 100 times. Expression of the *apvRep* genes of CNPV and FPV was assessed using publicly available data (PRJNA524335) (36). RNA-Seq reads were mapped to respective reference genomes (NC_005309 and AJ581527) using Burrows-Wheeler Aligner (BWA) (78), and coverages across *apvRep* genes were plotted.

### Phylogenetic Analysis of the *Poxviridae* and *Draupnirviridae*.

Protein sequences of nine conserved genes (major core protein 4a, major core protein 4b, DNA polymerase, RNA polymerase subunit RPO132, RNA polymerase subunit RPO147, messenger RNA capping enzyme catalytic subunit, RNA polymerase-associated protein RAP94, early transcription factor large subunit, and primase D5) were extracted from representatives of all official or proposed genera in the *Poxviridae*. These were concatenated, aligned with MAFFT, and analyzed with IQ-TREE as above. We produced a second tree (not shown) using four core genes (RNA polymerase subunit RPO132, RNA polymerase subunit RPO147, early transcription factor large subunit, and RNA polymerase-associated protein RAP94), previously identified to produce phylogenies consistent with whole genome analyses (59), and this allowed inclusion of additional partial genomes for which all the nine genes were not available. Branch order was observed to be highly consistent between the trees. The same methods were applied to phylogenetic analysis of cressdnaviruses.

### Detection and Analysis of EVEs.

Two rounds of EVE discovery were performed using the HGT detection workflow described above. Round one used the same protein query and targeted 24,764 eukaryotic genome assemblies available in GenBank and RefSeq databases in July 2022. Curation of hits began with DIAMOND analysis, this time after removing stop codons. Putative EVEs in contigs <4 kb were discarded, as were those in assemblies apparently containing numerous cressdnaviral lineages, which raised suspicion of contamination. Round two used a protein query comprehensively covering draupnirviruses and any draupnirvirus-related EVEs from the first round. We now targeted 6,639 assemblies, with an inclusive focus on apparent host lineages of *Draupnirviridae* (e.g., Sauropsida, Insecta, and SAR). Quality control of candidate EVEs was as above. Cluster analysis of Cap sequences was done using CLANS (79). To determine homology between curated elements, sequence contexts were masked for EVE sequences using maskfasta within BEDTools v2.27.1 (65) and all-versus-all aligned using LAST v1422 (80). Self-alignments were removed, along with any alignment below 300 bp in length (query strand) or with e-value over 1e^−50^. To ensure cutoff suitability, manual curation of selected low scoring alignments was done using D-GENIES v1.4 (81), which was also used to produce alignment plots.

## Supplementary Material

Appendix 01 (PDF)

Appendix 02 (PDF)

## Data Availability

All genome assemblies and datasets analyzed here are available in public databases. The computational workflow for HGT detection is available at: https://github.com/CormacKinsella/HGT_finder (63). Predicted protein structures described here are available at: https://figshare.com/projects/RepStructures/158462 (82). All other data are included in the manuscript and/or *SI Appendix*.
