# Supplementary material for: Vertebrate-tropism of a cressdnavirus lineage implicated by poxvirus gene capture
Source: Proc Natl Acad Sci U S A. 2023 May 8;120(20):e2303844120. doi: 10.1073/pnas.2303844120 (PMC10193959; doi:10.1073/pnas.2303844120)
Supplement: Supplementary file 1 — Appendix 01 (PDF) [file pnas.2303844120.sapp1.pdf]

Supplementary figures for article:

Vertebrate-tropism of a cressdnavirus lineage implicated by poxvirus gene capture

Cormac M. Kinsella, Lia van der Hoek

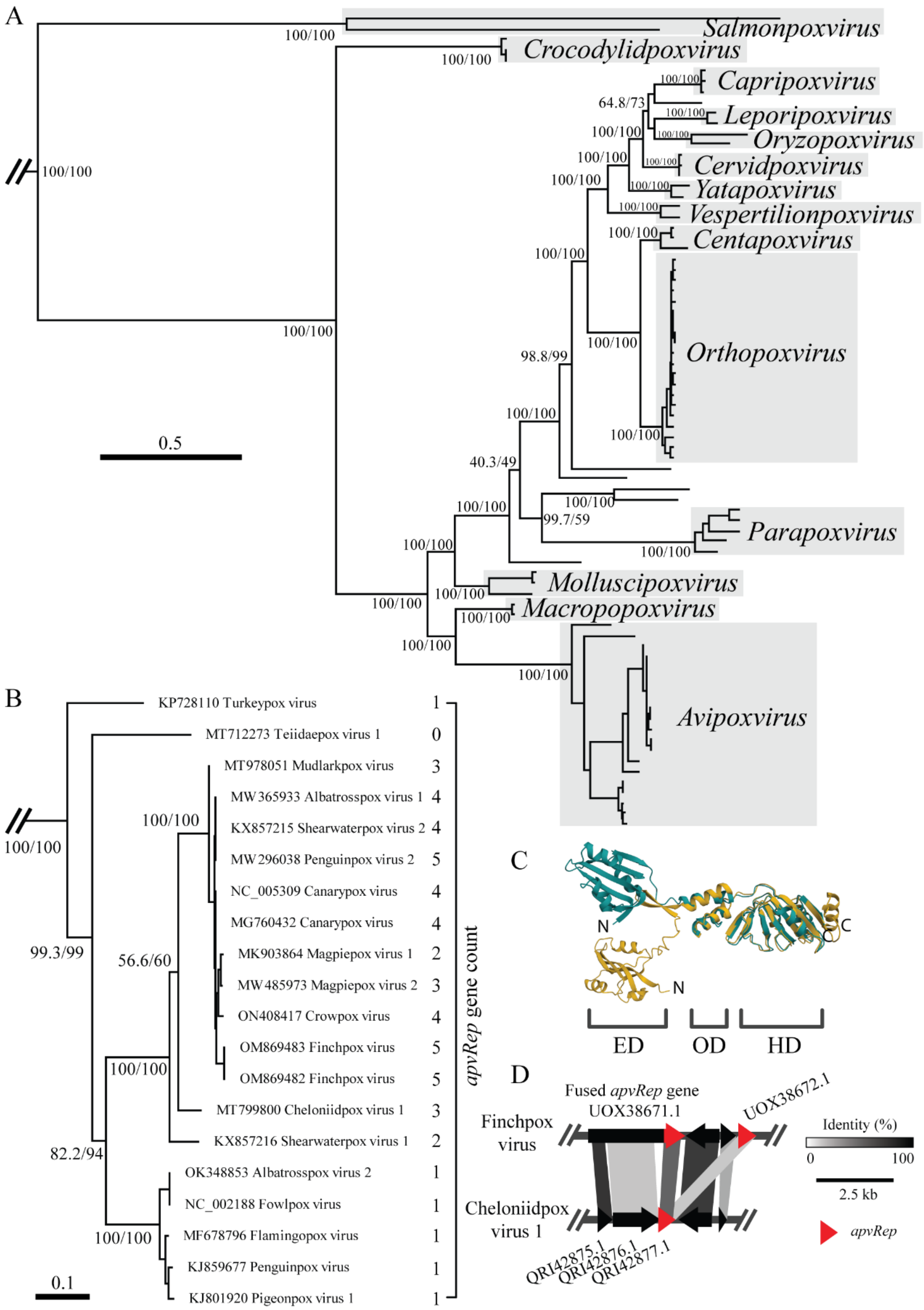

Supplementary Figure 1. A) Rooted maximum-likelihood phylogeny of the subfamily *Chordopoxvirinae*. Proposed genera with only one member are not labelled. Scale bar is in amino acid substitutions per site. Branch supports report SH-aLRT scores on the left and ultrafast bootstrap scores on the right. *Entomopoxvirinae* sequences were used for an outgroup (not shown). B) Focus on the genus *Avipoxvirus* within the same phylogeny. The *apvRep* gene count per-genome is shown at right. C) Superposed alignment between AlphaFold predicted structures of TM-6c Rep (cyan) and CNPV153 (gold) performed with the jFATCAT rigid algorithm; root-mean-square deviation = 1.61, TM-score = 0.53, query (TM-6c) coverage 65%, target coverage 55%. ED = endonuclease domain, OD = oligomerisation domain, HD = helicase domain. N and C denote the respective termini. D) Focused section of a synteny map between finchpox virus (OM869483.1) and cheloniidpox virus 1 (MT799800.1), revealing gene fusion has occurred in the former.

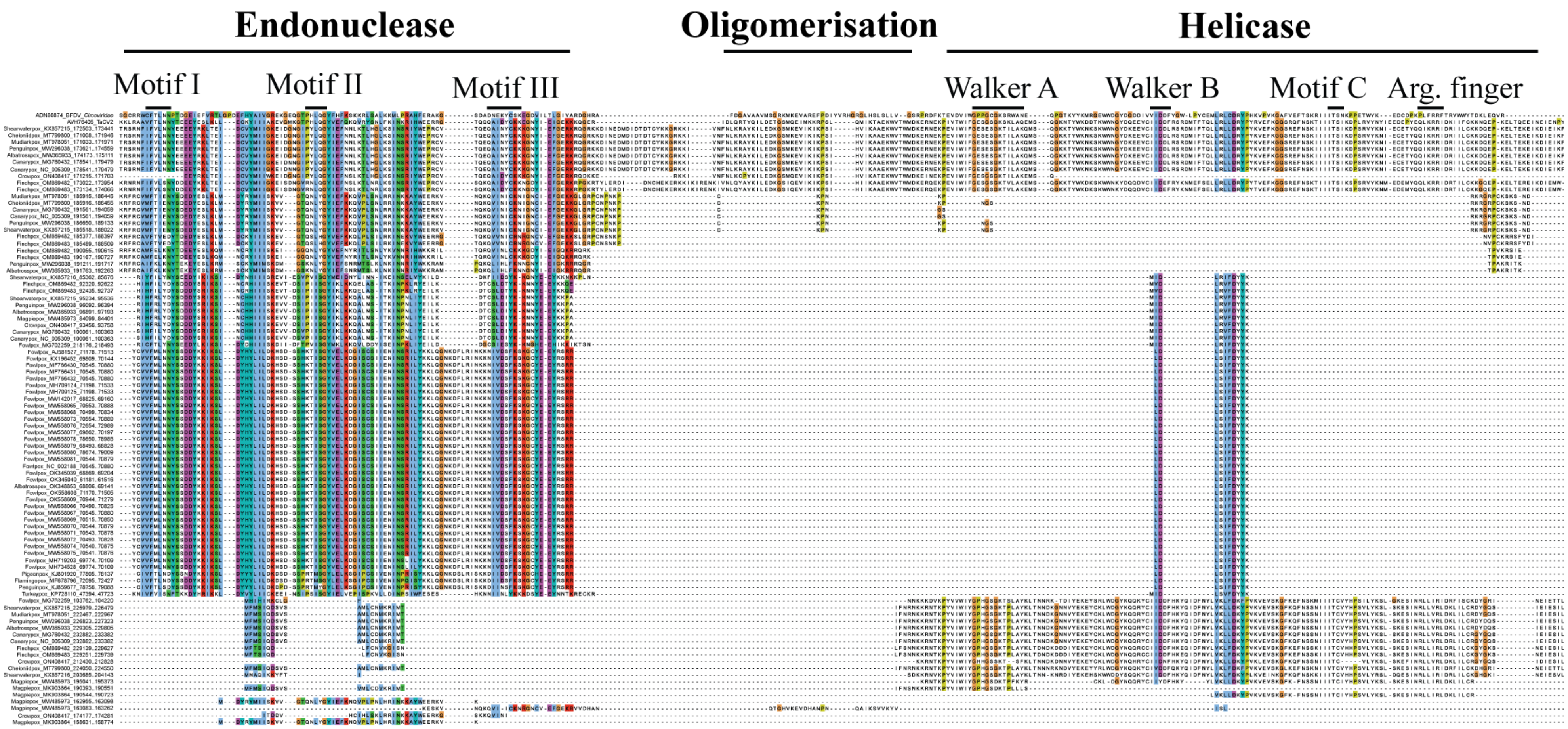

Supplementary Figure 2. Alignment between Reps of *Circoviridae*, *Krikovirus*, and predicted protein sequence of *apvRep* genes. Protein domains are annotated above.

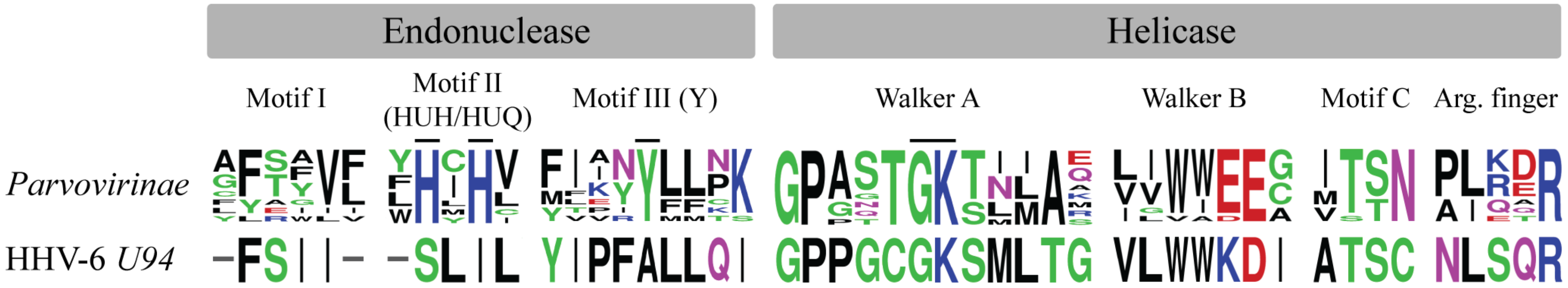

Supplementary Figure 3. Rep protein sequence motifs in the *Parvoviridae* (subfamily *Parvovirinae*), and the *U94* gene of human herpesvirus 6 (AVK93697.1). Arg. = arginine. Residue colours: hydrophobic = black, polar = green, basic = blue, acidic = red, neutral = purple. Key residues discussed in the main text are marked.

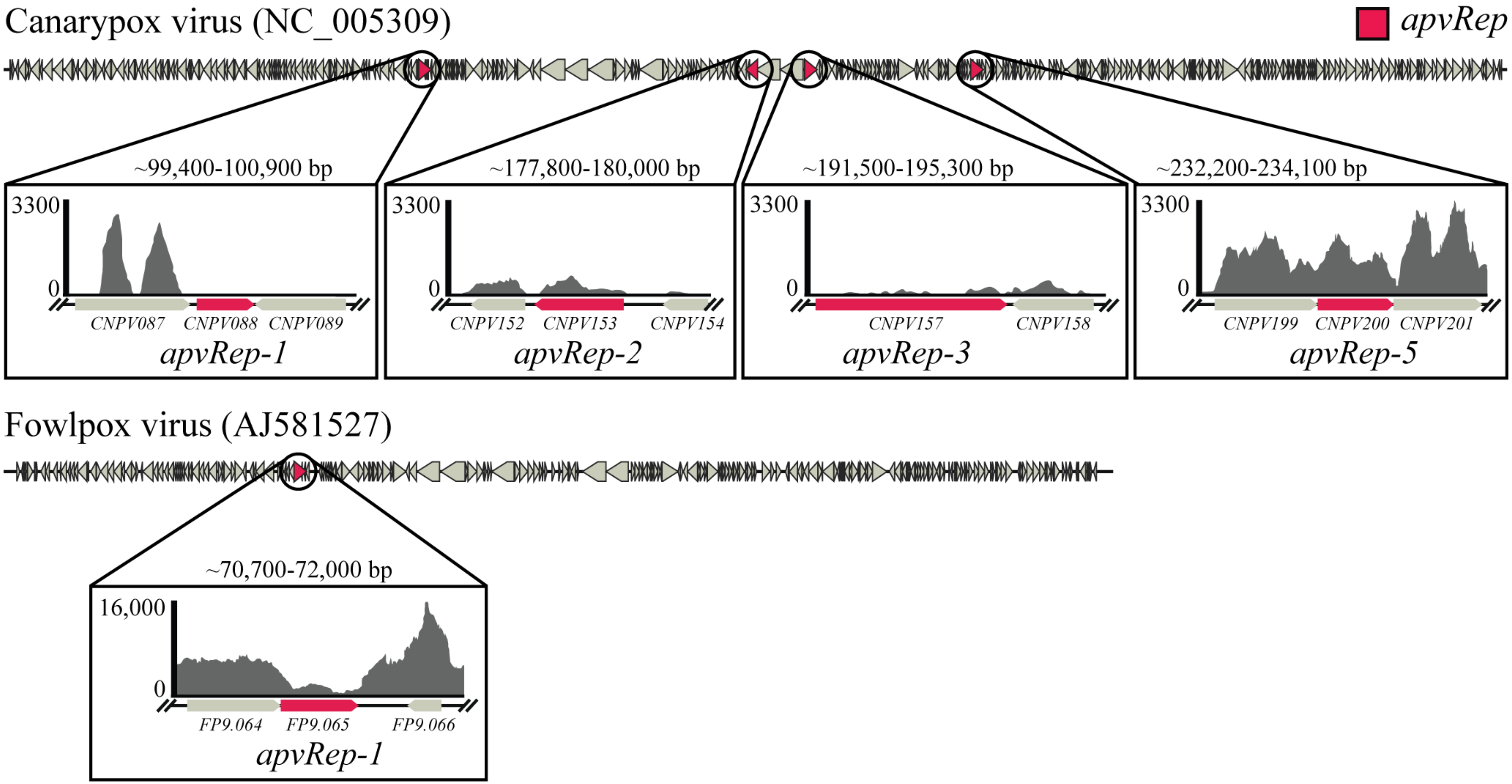

Supplementary Figure 4. Expression of *apvRep* genes in canarypox virus and fowlpox virus infected chicken embryonic stem cells. The y-axes show read coverage per site. At the 16-hour timepoint, there is no evidence of *apvRep-1* expression in canarypox virus, but other *apvRep* genes are expressed at different degrees. At 16-hours, *apvRep-1* is expressed by fowlpox virus.

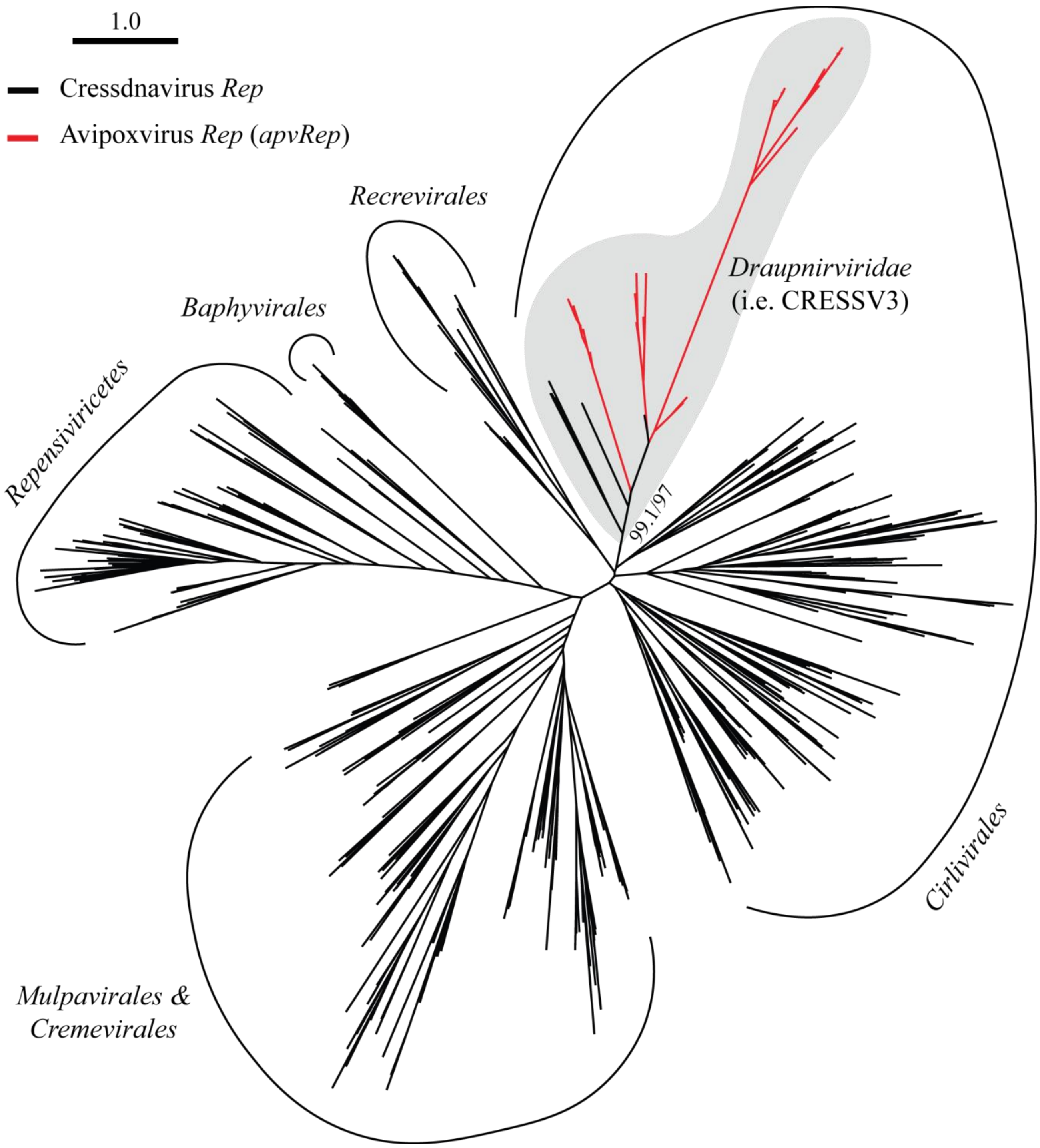

Supplementary Figure 5. Maximum-likelihood phylogeny of representative Rep sequences across the *Cressdnaviricota*, with the addition of *apvRep* predicted sequences. Scale bar is in amino acid substitutions per site. Branch support reports SH-aLRT scores on the left and ultrafast bootstrap scores on the right.

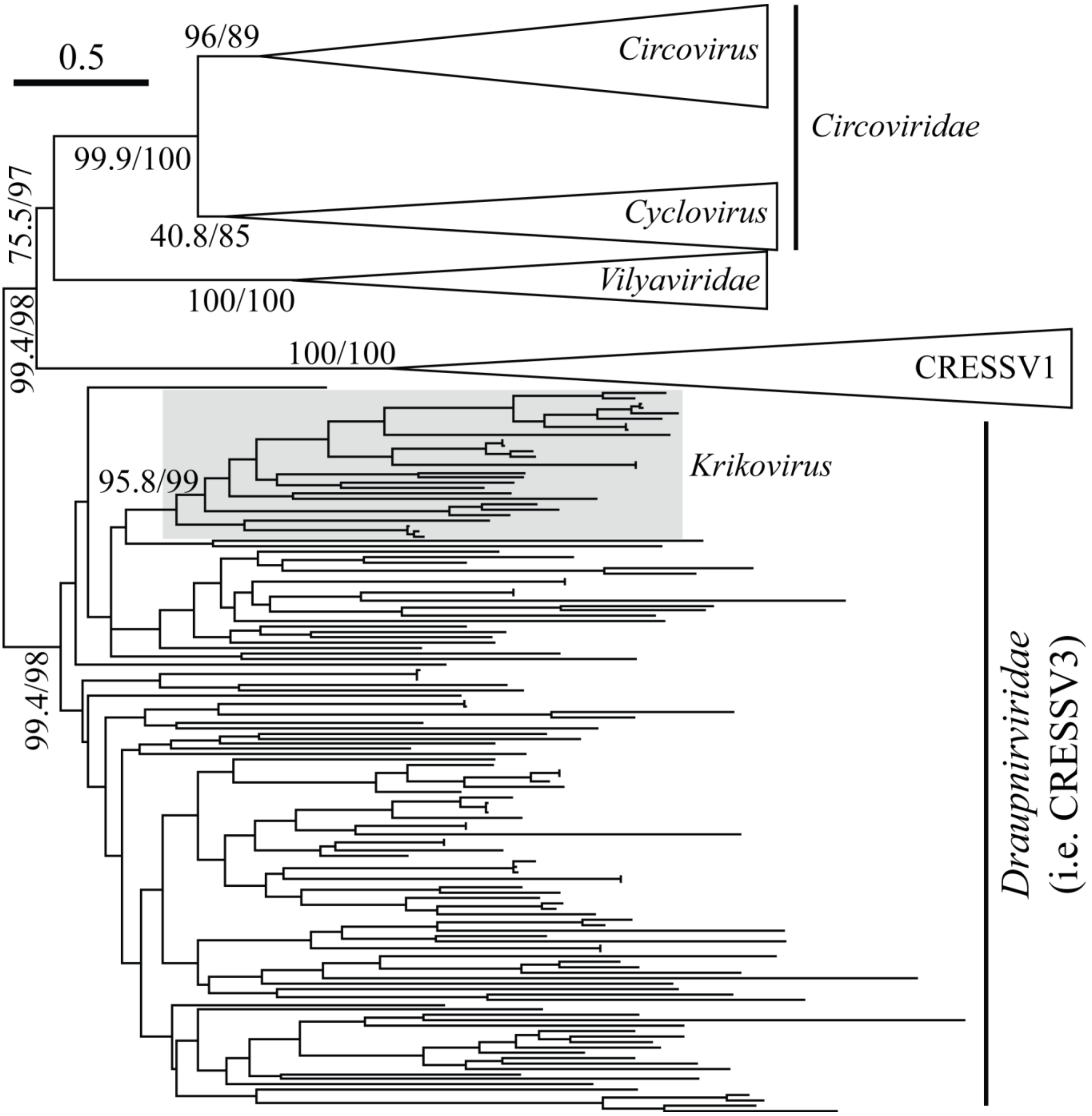

Supplementary Figure 6. Maximum-likelihood phylogeny of selected cressdnavirus Rep lineages. Scale bar is in amino acid substitutions per site. Branch supports report SH-aLRT scores on the left and ultrafast bootstrap scores on the right.

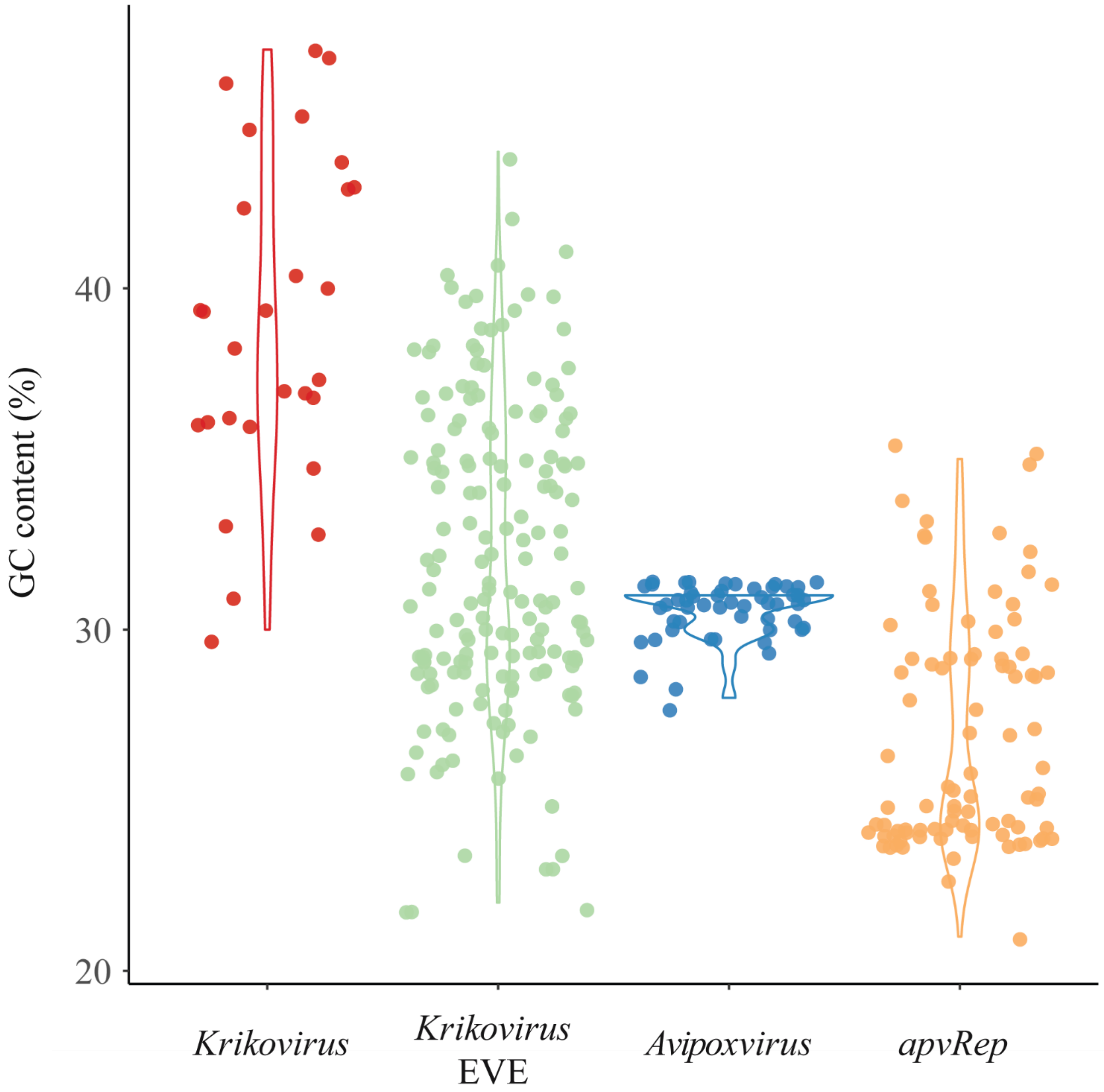

Supplementary Figure 7. GC contents of various sequences, including whole genomes of krikoviruses and avipoxviruses, krikovirus EVEs in animal genomes, and *apvRep* alleles in avipoxvirus genomes.
