# Supplementary material for: Vertebrate-tropism of a cressdnavirus lineage implicated by poxvirus gene capture
Source: Proc Natl Acad Sci U S A. 2023 May 8;120(20):e2303844120. doi: 10.1073/pnas.2303844120 (PMC10193959; doi:10.1073/pnas.2303844120)
Supplement: Supplementary file 2 — Appendix 02 (PDF) [file pnas.2303844120.sapp2.pdf]

Supplementary Table 1. *Avipoxvirus* genome assemblies examined as part of this study.

**Name**	**Accession**	***apvRep+***	**Genome length (kb)**
Albatrosspox virus 1 strain SAN97-0665NZ	MW365933	Yes	352
Albatrosspox virus 2 strain TST/1997	OK348853	Yes	286
Canarypox virus isolate FMV15SnBuPox	MG760432	Yes	359
Canarypox virus strain ATCC VR-111	NC_005309	Yes	365
Cheloniid poxvirus 1 strain AUS	MT799800	Yes	343
Crowpox virus strain 122740AU/2021	ON408417	Yes	329
Finch poxvirus isolate pox_34731	OM869482	Yes	354
Finch poxvirus isolate pox_35627	OM869483	Yes	354
Flamingopox virus KD09	MF678796	Yes	293
Fowlpox virus (FPVUS)	NC_002188	Yes	288
Fowlpox virus isolate 16055_trachea_170512	MF766430	Yes	289
Fowlpox virus isolate 16069_trachea_170323	MF766431	Yes	289
Fowlpox virus isolate 16117_scab_170512	MF766432	Yes	289
Fowlpox virus isolate 282E4	MG702259	Yes	309
Fowlpox virus isolate HP-438/Munich, passage 438 clone FP9	AJ581527	Yes	266
Fowlpox virus isolate PA18/24608	OK345039	Yes	263
Fowlpox virus isolate PA19/1236	OK345040	Yes	283
Fowlpox virus MN00.1	MH709125	Yes	291
Fowlpox virus MN00.2	MH709124	Yes	299
Fowlpox virus SD15-670.1	MH719203	Yes	288
Fowlpox virus SD15-670.2	MH734528	Yes	296
Fowlpox virus strain 05113	MW558074	Yes	297
Fowlpox virus strain 10D392	MW558065	Yes	297
Fowlpox virus strain 13D121	MW558066	Yes	297
Fowlpox virus strain 14D047	MW558067	Yes	297
Fowlpox virus strain 15D039	MW558068	Yes	297

Fowlpox virus strain 18Q061	MW558069	Yes	297
Fowlpox virus strain 18R056	MW558070	Yes	297
Fowlpox virus strain 18R059	MW558071	Yes	297
Fowlpox virus strain 19D064	MW558072	Yes	297
Fowlpox virus strain 2755	MW558073	Yes	297
Fowlpox virus strain 99866	MW558075	Yes	297
Fowlpox virus strain FWPV-S (fowlpox virus vaccine strain)	MW142017	Yes	294
Fowlpox virus strain NX10	KX196452	Yes	282
Fowlpox virus strain V_blen	MW558077	Yes	291
Fowlpox virus strain V_cmp	MW558078	Yes	287
Fowlpox virus strain V_ds	MW558079	Yes	278
Fowlpox virus strain V_ja	MW558081	Yes	288
Fowlpox virus strain V_kr	MW558080	Yes	292
Fowlpox virus strain V_poxine	MW558076	Yes	296
Fowlpox virus strain W775-18 isolate FPV-CAMs clone e4-dwmau9f	OK558608	Yes	299
Fowlpox virus strain W775-18 isolate FPV-COMB	OK558609	Yes	298
Magpiepox virus 1	MK903864	Yes	293
Magpiepox virus 2	MW485973	Yes	298
Mudlarkpox virus strain MLPV-AU2019	MT978051	Yes	343
Penguinpox virus 2 strain YEP-NZ	MW296038	Yes	350
Penguinpox virus isolate PSan92	KJ859677	Yes	306
Pigeonpox virus 1 isolate FeP2	KJ801920	Yes	282
Shearwaterpox virus strain SWPV-1	KX857216	Yes	327
Shearwaterpox virus strain SWPV-2	KX857215	Yes	351
Teiidaepox virus 1 isolate 1642/19	MT712273	No	167
Turkeypox virus strain TKPV-HU1124/2011	KP728110	Yes	188.5

Supplementary Table 2. Detected *apvRep* sequences.

**Virus name**	**Virus accession**	***apvRep* gene count in assembly**	***apvRep* accession**	**Coordinates**	**Length (aa)**	**Domains**	**Gene**	**Notes**
Albatrosspox virus 1 strain SAN97-0665NZ	MW365933	4	QRM16053	96891..97193	100	endonuclease	apvRep-1	
Albatrosspox virus 2 strain TST/1997	OK348853	1	UNS14260	68806..69141	111	endonuclease	apvRep-1	
Canarypox virus isolate FMV15SnBuPox	MG760432	4	AWD84564	100061..100363	100	endonuclease	apvRep-1	
Canarypox virus strain ATCC VR-111	NC_005309	4	NP_955111	100061..100363	100	endonuclease	apvRep-1	
Crowpox virus strain 122740AU/2021	ON408417	4	UWX11229	93456..93758	100	endonuclease	apvRep-1	
Finch poxvirus isolate pox_34731	OM869482	5	UOX38947	92320..92622	100	endonuclease	apvRep-1	
Finch poxvirus isolate pox_35627	OM869483	5	UOX38613	92435..92737	100	endonuclease	apvRep-1	
Flamingopox virus KD09	MF678796	1	AUD40167	72095..72427	110	endonuclease	apvRep-1	
Fowlpox virus (FPVUS)	NC_002188	1	NP_039028	70545..70880	111	endonuclease	apvRep-1	
Fowlpox virus isolate 16055_trachea_170512	MF766430	1	AXY04507	70545..70880	111	endonuclease	apvRep-1	
Fowlpox virus isolate 16069_trachea_170323	MF766431	1	AXY04769	70545..70880	111	endonuclease	apvRep-1	
Fowlpox virus isolate 16117_scab_170512	MF766432	1	AXY05028	70545..70880	111	endonuclease	apvRep-1	
Fowlpox virus isolate 282E4	MG702259	2	AYP74236	218176..218493	105	endonuclease	apvRep-1	assembly is reversed
Fowlpox virus isolate HP-438/Munich, passage 438 clone FP9	AJ581527	1	CAE52610	71178..71513	111	endonuclease	apvRep-1	
Fowlpox virus isolate PA18/24608	OK345039	1	UQT20360	68869..69204	111	endonuclease	apvRep-1	

Fowlpox virus isolate PA19/1236	OK345040	1	UQT20601	61181..61516	111	endonuclease	apvRep-1	
Fowlpox virus MN00.1	MH709125	1	AYO89918	71198..71533	111	endonuclease	apvRep-1	
Fowlpox virus MN00.2	MH709124	1	AYO89659	71198..71533	111	endonuclease	apvRep-1	
Fowlpox virus SD15-670.1	MH719203	1	AYO90177	69774..70109	111	endonuclease	apvRep-1	
Fowlpox virus SD15-670.2	MH734528	1	AYO90434	69774..70109	111	endonuclease	apvRep-1	
Fowlpox virus strain 05113	MW558074	1	URH27141	70540..70875	111	endonuclease	apvRep-1	
Fowlpox virus strain 10D392	MW558065	1	URH24784	70553..70888	111	endonuclease	apvRep-1	
Fowlpox virus strain 13D121	MW558066	1	URH25042	70490..70825	111	endonuclease	apvRep-1	
Fowlpox virus strain 14D047	MW558067	1	URH25306	70545..70880	111	endonuclease	apvRep-1	
Fowlpox virus strain 15D039	MW558068	1	URH25567	70499..70834	111	endonuclease	apvRep-1	
Fowlpox virus strain 18Q061	MW558069	1	URH25825	70515..70850	111	endonuclease	apvRep-1	
Fowlpox virus strain 18R056	MW558070	1	URH26089	70544..70879	111	endonuclease	apvRep-1	
Fowlpox virus strain 18R059	MW558071	1	URH26352	70543..70878	111	endonuclease	apvRep-1	
Fowlpox virus strain 19D064	MW558072	1	URH26614	70493..70828	111	endonuclease	apvRep-1	
Fowlpox virus strain 2755	MW558073	1	URH26879	70554..70889	111	endonuclease	apvRep-1	
Fowlpox virus strain 99866	MW558075	1	URH27405	70541..70876	111	endonuclease	apvRep-1	
Fowlpox virus strain FWPV-S (fowlpox virus vaccine strain)	MW142017	1	QRM13602	68825..69160	111	endonuclease	apvRep-1	
Fowlpox virus strain NX10	KX196452	1	ART91499	69809..70144	111	endonuclease	apvRep-1	
Fowlpox virus strain V_blen	MW558077	1	URH27931	69862..70197	111	endonuclease	apvRep-1	
Fowlpox virus strain V_cmp	MW558078	1	URH28187	78650..78985	111	endonuclease	apvRep-1	
Fowlpox virus strain V_ds	MW558079	1	URH28446	68493..68828	111	endonuclease	apvRep-1	
Fowlpox virus strain V_ja	MW558081	1	URH28966	70544..70879	111	endonuclease	apvRep-1	
Fowlpox virus strain V_kr	MW558080	1	URH28705	78674..79009	111	endonuclease	apvRep-1	
Fowlpox virus strain V_poxine	MW558076	1	URH27669	72654..72989	111	endonuclease	apvRep-1	
Fowlpox virus strain W775-18 isolate FPV-CAMs clone e4-dwmau9f	OK558608	1	UHJ15129	71170..71505	111	endonuclease	apvRep-1	
Fowlpox virus strain W775-18 isolate FPV-COMB	OK558609	1	UHJ15386	70944..71279	111	endonuclease	apvRep-1	

Magpiepox virus 2	MW485973	3	QZW33388	84099..84401	100	endonuclease	apvRep-1	
Penguinpox virus 2 strain YEP-NZ	MW296038	5	QRM15721	96092..96394	100	endonuclease	apvRep-1	
Penguinpox virus isolate PSan92	KJ859677	1	AID46805	78756..79088	110	endonuclease	apvRep-1	
Pigeonpox virus 1 isolate FeP2	KJ801920	1	AID46576	77805..78137	110	endonuclease	apvRep-1	
Shearwaterpox virus strain SWPV-1	KX857216	2	ARE67659	85362..85676	104	endonuclease	apvRep-1	
Shearwaterpox virus strain SWPV-2	KX857215	4	ARE67306	95234..95536	100	endonuclease	apvRep-1	
Turkeypox virus strain TKPV-HU1124/2011	KP728110	1	ALA62414	47394..47723	109	endonuclease	apvRep-1	
Albatrosspox virus 1 strain SAN97-0665NZ	MW365933	4	QRM16118	174173..175111	312	all domains	apvRep-2	
Canarypox virus isolate FMV15SnBuPox	MG760432	4	AWD84629	178541..179479	312	all domains	apvRep-2	
Canarypox virus strain ATCC VR-111	NC_005309	4	NP_955176	178541..179479	312	all domains	apvRep-2	CNPV153
Cheloniid poxvirus 1 strain AUS	MT799800	3	QRI42871	171008..171946	312	all domains	apvRep-2	
Crowpox virus strain 122740AU/2021	ON408417	4	UWX11309	171215..171703	162	endonuclease	apvRep-2	degraded
Finch poxvirus isolate pox_34731	OM869482	5	UOX39153	173022..173954	310	all domains	apvRep-2	
Finch poxvirus isolate pox_35627	OM869483	5	UOX38818	173134..174066	310	all domains	apvRep-2	
Mudlarkpox virus strain MLPV-AU2019	MT978051	3	QRM15430	171033..171971	312	all domains	apvRep-2	
Penguinpox virus 2 strain YEP-NZ	MW296038	5	QRM15786	173621..174559	312	all domains	apvRep-2	
Shearwaterpox virus strain SWPV-2	KX857215	4	ARE67375	172503..173441	312	all domains	apvRep-2	
Canarypox virus isolate FMV15SnBuPox	MG760432	4	AWD84632	191561..194059	832	endonuclease	apvRep-3	gene fusion

Canarypox virus strain ATCC VR-111	NC_005309	4	NP_955179	191561..194059	832	endonuclease	apvRep-3	gene fusion
Cheloniid poxvirus 1 strain AUS	MT799800	3	QRI42877	185916..186455	179	endonuclease	apvRep-3	
Crowpox virus strain 122740AU/2021	ON408417	4	NA	174177..174281	35	endonuclease	apvRep-3	degraded
Finch poxvirus isolate pox_34731	OM869482	5	UOX39006	185377..188397	1006	endonuclease	apvRep-3	gene fusion
Finch poxvirus isolate pox_35627	OM869483	5	UOX38671	185489..188509	1006	endonuclease	apvRep-3	gene fusion
Magpiepox virus 1	MK903864	2	QGM48791	158631..158774	47	endonuclease	apvRep-3	degraded
Magpiepox virus 2	MW485973	3	QZW33470	162955..163098	47	endonuclease	apvRep-3	degraded, fragment 1
Magpiepox virus 2	MW485973	3	NA	163083..163262	59	endonuclease	apvRep-3	degraded, fragment 2
Mudlarkpox virus strain MLPV-AU2019	MT978051	3	QRM15435	185915..186445	176	endonuclease	apvRep-3	
Penguinpox virus 2 strain YEP-NZ	MW296038	5	QRM15789	186650..189133	827	endonuclease	apvRep-3	gene fusion
Shearwaterpox virus strain SWPV-2	KX857215	4	ARE67380	185518..188022	834	endonuclease	apvRep-3	gene fusion
Albatrosspox virus 1 strain SAN97-0665NZ	MW365933	4	QRM16124	191763..192263	166	endonuclease	apvRep-4	
Finch poxvirus isolate pox_34731	OM869482	5	UOX39007	190055..190615	186	endonuclease	apvRep-4	
Finch poxvirus isolate pox_35627	OM869483	5	UOX38672	190167..190727	186	endonuclease	apvRep-4	
Penguinpox virus 2 strain YEP-NZ	MW296038	5	QRM15792	191211..191717	168	endonuclease	apvRep-4	
Albatrosspox virus 1 strain SAN97-0665NZ	MW365933	4	QRM16166	229305..229805	166	helicase	apvRep-5	
Canarypox virus isolate FMV15SnBuPox	MG760432	4	AWD84676	232882..233382	166	helicase	apvRep-5	
Canarypox virus strain ATCC	NC_005309	4	NP_955223	232882..233382	166	helicase	apvRep-5	CNPV200

VR-111								
Cheloniid poxvirus 1 strain AUS	MT799800	3	QRI42919	224050..224550	166	helicase	apvRep-5	
Crowpox virus strain 122740AU/2021	ON408417	4	NA	212430..212828	133	helicase	apvRep-5	degraded
Finch poxvirus isolate pox_34731	OM869482	5	UOX39008	229139..229627	162	helicase	apvRep-5	
Finch poxvirus isolate pox_35627	OM869483	5	UOX38673	229251..229739	162	helicase	apvRep-5	
Fowlpox virus isolate 282E4	MG702259	2	AYP74145	103762..104220	152	helicase	apvRep-5	assembly is reversed
Magpiepox virus 1	MK903864	2	QGM48834	190393..190551	52	helicase	apvRep-5	degraded, fragment 1
Magpiepox virus 1	MK903864	2	NA	190544..190723	59	helicase	apvRep-5	degraded, fragment 2
Magpiepox virus 2	MW485973	3	NA	195041..195373	105	helicase	apvRep-5	degraded
Mudlarkpox virus strain MLPV-AU2019	MT978051	3	QRM15476	222467..222967	166	helicase	apvRep-5	
Penguinpox virus 2 strain YEP-NZ	MW296038	5	QRM15831	226823..227323	166	helicase	apvRep-5	
Shearwaterpox virus strain SWPV-1	KX857216	2	ARF02751	203685..204143	152	helicase	apvRep-5	
Shearwaterpox virus strain SWPV-2	KX857215	4	ARE67427	225979..226479	166	helicase	apvRep-5	

Supplementary Table 3. *Draupnirviridae* reference table.

**Family**	**Genus**	**Accession**	**Isolation source**
Draupnirviridae	Krikovirus	MF804498	avipoxvirus-induced lesion on bird
Draupnirviridae	Krikovirus	KY302864	bat feces
Draupnirviridae	Krikovirus	JN857329	bat feces
Draupnirviridae	Krikovirus	JN377580	bat feces
Draupnirviridae	Krikovirus	KY302869	bat feces
Draupnirviridae	Krikovirus	KM382272	bat feces
Draupnirviridae	Krikovirus	KM382271	bat feces
Draupnirviridae	Krikovirus	HM228875	bat guano
Draupnirviridae	Krikovirus	MT734814	bat guano samples
Draupnirviridae	Krikovirus	MT734815	bat guano samples
Draupnirviridae	Krikovirus	MT734813	bat guano samples
Draupnirviridae	Krikovirus	OL704834	bat-associated
Draupnirviridae	Krikovirus	KJ641718	bat-associated
Draupnirviridae	Krikovirus	KJ641729	bat-associated
Draupnirviridae	Krikovirus	KJ641722	bat-associated
Draupnirviridae	Krikovirus	MW588080	bird (swan stool)
Draupnirviridae	Krikovirus	MT138040	bird anal swab
Draupnirviridae	Krikovirus	MT138069	bird anal swab
Draupnirviridae	Krikovirus	MN928934	bird anal swab
Draupnirviridae	Krikovirus	MW182733	bird anal swab
Draupnirviridae	Krikovirus	KF738883	dragonfly
Draupnirviridae	Krikovirus	NC_023435	dragonfly
Draupnirviridae	Krikovirus	HQ335042	mosquito
Draupnirviridae	Krikovirus	NC_026512	mosquito (Aedes vexans)
Draupnirviridae	Krikovirus	KM972726	mosquito (Aedes vexans)
Draupnirviridae	Krikovirus	OL700204	mosquito (Culex)

Draupnirviridae	Krikovirus	NC_040576	mosquito (Culex)
Draupnirviridae	Krikovirus	MH188041	mosquito (Culex)
Draupnirviridae		MW678877	airborne particulate matter
Draupnirviridae		MW678988	airborne particulate matter
Draupnirviridae		NC_026628	Avon-Heathcote Estuary associated
Draupnirviridae		KX828614	Barents Sea sediment
Draupnirviridae		MT671990	beef
Draupnirviridae		NC_026635	benthic sediment
Draupnirviridae		MW182747	bird anal swab
Draupnirviridae		MT138072	bird anal swab
Draupnirviridae		MW182844	bird anal swab
Draupnirviridae		MT138045	bird anal swab
Draupnirviridae		MN928938	bird anal swab
Draupnirviridae		MN928937	bird anal swab
Draupnirviridae		MW182755	bird anal swab
Draupnirviridae		MW182819	bird anal swab
Draupnirviridae		MW182736	bird anal swab
Draupnirviridae		KY312555	bird stool
Draupnirviridae		KP153422	Chironomus zealandicus (non-biting midge)
Draupnirviridae		MH617295	cow tissue
Draupnirviridae		MK012433	crucian tissue
Draupnirviridae		MK012508	crucian tissue
Draupnirviridae		BAKC01000020	deep-sea sediment off the Shimokita Peninsula
Draupnirviridae		BAKC01000070	deep-sea sediment off the Shimokita Peninsula
Draupnirviridae		BAKC01000006	deep-sea sediment off the Shimokita Peninsula
Draupnirviridae		KM598404	dragonfly
Draupnirviridae		NC_040527	dragonfly
Draupnirviridae		JX185418	dragonfly
Draupnirviridae		NC_023855	dragonfly
Draupnirviridae		KM598406	dragonfly

Draupnirviridae		NC_029596	Echyridella menziesii (mussel)
Draupnirviridae		KT149403	Freshwater lake (Dianchi) sample (< 0.5 m)
Draupnirviridae		NC_030460	Freshwater lake (Dianchi) sample (< 0.5 m)
Draupnirviridae		KT149410	Freshwater lake (Donghu) sample (< 0.5 m)
Draupnirviridae		KT149409	Freshwater lake (Donghu) sample (< 0.5 m)
Draupnirviridae		NC_030477	Freshwater lake (Donghu) sample (< 0.5 m)
Draupnirviridae		MH617523	haddock tissue
Draupnirviridae		MH617702	haddock tissue
Draupnirviridae		BK029143	Human metagenome
Draupnirviridae		MW697482	Lake water sample
Draupnirviridae		MW697472	Lake water sample
Draupnirviridae		MW697478	Lake water sample
Draupnirviridae		MW202764	Manatee Spring
Draupnirviridae		MW202735	Manatee Spring
Draupnirviridae		MW202677	Manatee Spring
Draupnirviridae		MW202551	Manatee Spring
Draupnirviridae		MW202490	Manatee Spring
Draupnirviridae		MT181546	marmot stool
Draupnirviridae		MT181527	marmot stool
Draupnirviridae		NC_026632	mussel/estuary
Draupnirviridae		KM874305	mussel/estuary
Draupnirviridae		KM874304	mussel/estuary
Draupnirviridae		KM874294	mussel/estuary
Draupnirviridae		NC_026653	mussel/estuary
Draupnirviridae		KM874300	mussel/estuary
Draupnirviridae		KX388512	peat
Draupnirviridae		MT263533	plant
Draupnirviridae		KP153423	Potamopyrgus antipodarum (snail)
Draupnirviridae		NC_013026	reclaimed water
Draupnirviridae		MH616644	red snapper tissue

Draupnirviridae		MH617406	red snapper tissue
Draupnirviridae		MH616869	red snapper tissue
Draupnirviridae		MH617326	red snapper tissue
Draupnirviridae		MH617708	red snapper tissue
Draupnirviridae		JX904581	Saanich Inlet, British Columbia, Canada
Draupnirviridae		JX904407	Saanich Inlet, British Columbia, Canada
Draupnirviridae		KX246262	sea cucumber
Draupnirviridae		MH649188	seabass tissue
Draupnirviridae		MH648972	seabass tissue
Draupnirviridae		MH617385	seabass tissue
Draupnirviridae		MH649231	seabass tissue
Draupnirviridae		MH649090	seabass tissue
Draupnirviridae		KP153408	sediment
Draupnirviridae		NC_029589	sediment
Draupnirviridae		MT263571	sediments
Draupnirviridae		MT263551	sediments
Draupnirviridae		KM874317	snail associated
Draupnirviridae		JX904139	Strait of Georgia, British Columbia, Canada
Draupnirviridae		JX904076	Strait of Georgia, British Columbia, Canada
Draupnirviridae		JX904075	Strait of Georgia, British Columbia, Canada
Draupnirviridae		MW588081	swan stool
Draupnirviridae		KY487893	wastewater
Draupnirviridae		KY487838	wastewater
Draupnirviridae		KY487945	wastewater
Draupnirviridae		KY487847	wastewater
Draupnirviridae		KY487854	wastewater
Draupnirviridae		KY487866	wastewater
Draupnirviridae		KY487898	wastewater
Draupnirviridae		MT478520	water
Draupnirviridae		MT263655	water

Draupnirviridae		MT478494	water
Draupnirviridae		MT478518	water
Draupnirviridae		MT478521	water
Draupnirviridae		MT478527	water
Draupnirviridae		MT263594	water
Draupnirviridae		MT478457	water
Draupnirviridae		MT478550	water
Draupnirviridae		MT478531	water
Draupnirviridae		MT478555	water
Draupnirviridae		MT478515	water
Draupnirviridae		MT263553	water
Draupnirviridae		MT478560	water
Draupnirviridae		MT263566	water
Draupnirviridae		MK679543	water
Draupnirviridae		MT478556	water
Draupnirviridae		MT478483	water
Draupnirviridae		MT478552	water
Draupnirviridae		MT478506	water
Draupnirviridae		MW347483	water samples from river ports along the Yangtze River in China
Draupnirviridae		MW347384	water samples from river ports along the Yangtze River in China
Draupnirviridae		MW347500	water samples from river ports along the Yangtze River in China

Supplementary Table 4. Endogenous viral elements related to the *Draupnirviridae*.

Species	Assembly	Contig	Coordinates	Chromosome	Gene	Assignment	Homology group	Lowest shared taxon
Polymyxa betae	GCA_003693705.1	RBZT01000513.1	41484-41651	chrUnk	rep	Draupnirviridae	Draupnirviridae.044	Polymyxa_betae
Anilios bituberculatus	GCA_022379055.1	JAJFZI010162911.1	3236-3298	chrUnk	rep	Draupnirviridae	Draupnirviridae.045	Anilios_bituberculatus
Anilios bituberculatus	GCA_022379055.1	JAJFZI010169289.1	4173-4319	chrUnk	rep	Draupnirviridae	Draupnirviridae.046	Anilios_bituberculatus
Chromera velia	GCA_018398765.1	JAHBMJ010000396.1	3824-4792	chrUnk	rep	Draupnirviridae	Draupnirviridae.047	Chromera_velia
Chromera velia	GCA_000585135.1	ARZB01032935.1	399-1257	chrUnk	rep	Draupnirviridae	Draupnirviridae.047	Chromera_velia
Chromera velia	GCA_018398765.1	JAHBMJ010004271.1	8511-9369	chrUnk	rep	Draupnirviridae	Draupnirviridae.047	Chromera_velia
Chromera velia	GCA_018398765.1	JAHBMJ010003647.1	71382-71993	chrUnk	rep	Draupnirviridae	Draupnirviridae.047	Chromera_velia
Chromera velia	GCA_018398765.1	JAHBMJ010003647.1	72441-72888	chrUnk	rep	Draupnirviridae	Draupnirviridae.047	Chromera_velia
Chromera velia	GCA_018398765.1	JAHBMJ010004305.1	54572-55261	chrUnk	rep	Draupnirviridae	Draupnirviridae.048	Chromera_velia
Chromera velia	GCA_018398765.1	JAHBMJ010004305.1	53307-53563	chrUnk	rep	Draupnirviridae	Draupnirviridae.048	Chromera_velia
Chromera velia	GCA_018398765.1	JAHBMJ010002362.1	15794-16673	chrUnk	rep	Draupnirviridae	Draupnirviridae.049	Chromera_velia
Arizona elegans	GCA_022577455.1	JAKOOJ010000001.1	56659389-56659577	chr1	rep	Draupnirviridae_Krikovirus	Draupnirviridae-Krikovirus.001	Serpentes
Arizona elegans	GCA_022578425.1	JAKOOK010012251.1	13215-13403	chr1	rep	Draupnirviridae_Krikovirus	Draupnirviridae-Krikovirus.001	Serpentes

Azemiops feae	GCA_023970755.1	JALGBA010003977.1	547470-547655	chr1	rep	Draupnirviridae_Krikovirus	Draupnirviridae-Krikovirus.001	Serpentes
Bothrops jararaca	GCA_018340635.1	JAGTXL010020801.1	319135-319320	chr1	rep	Draupnirviridae_Krikovirus	Draupnirviridae-Krikovirus.001	Serpentes
Bungarus multicinctus	GCA_023653725.1	CM042838.1	100758776-100758960	chr1	rep	Draupnirviridae_Krikovirus	Draupnirviridae-Krikovirus.001	Serpentes
Charina bottae	GCA_023362785.1	JALMGK010000002.1	72180968-72181147	chr1	rep	Draupnirviridae_Krikovirus	Draupnirviridae-Krikovirus.001	Serpentes
Charina bottae	GCA_023362775.1	JALMGJ010000001.1	150358822-150358989	chr1	rep	Draupnirviridae_Krikovirus	Draupnirviridae-Krikovirus.001	Serpentes
Chrysopelea ornata	GCA_019457695.1	JAHWGE011116266.1	3269-3403	chr1	rep	Draupnirviridae_Krikovirus	Draupnirviridae-Krikovirus.001	Serpentes
Crotalus adamanteus	GCA_018446365.1	JADPQB010002565.1	593883-594068	chr1	rep	Draupnirviridae_Krikovirus	Draupnirviridae-Krikovirus.001	Serpentes
Crotalus horridus	GCA_001625485.1	LVCR01045672.1	7533-7718	chr1	rep	Draupnirviridae_Krikovirus	Draupnirviridae-Krikovirus.001	Serpentes
Crotalus oreganus	GCA_024509115.1	JAKSXO010000001.1	64179136-64179321	chr1	rep	Draupnirviridae_Krikovirus	Draupnirviridae-Krikovirus.001	Serpentes
Crotalus oreganus	GCA_024509165.1	JAKSXP010000001.1	140951064-140951249	chr1	rep	Draupnirviridae_Krikovirus	Draupnirviridae-Krikovirus.001	Serpentes
Crotalus tigris	GCF_016545835.1	NW_024100515.1	992859-993044	chr1	rep	Draupnirviridae_Krikovirus	Draupnirviridae-Krikovirus.001	Serpentes
Crotalus tigris	GCA_016545835.1	VORL01003840.1	992859-993044	chr1	rep	Draupnirviridae_Krikovirus	Draupnirviridae-Krikovirus.001	Serpentes

Crotalus viridis	GCA_003400415.2	CM012306.1	60478747-60478932	chr1	rep	Draupnirviridae_Krikovirus	Draupnirviridae-Krikovirus.001	Serpentes
Daboia siamensis	GCA_024449315.1	JAKJMR010000114.1	41235-41423	chr1	rep	Draupnirviridae_Krikovirus	Draupnirviridae-Krikovirus.001	Serpentes
Diadophis punctatus	GCA_023053685.1	JALIGW010000001.1	264421059-264421247	chr1	rep	Draupnirviridae_Krikovirus	Draupnirviridae-Krikovirus.001	Serpentes
Emydocephalus ijimae	GCA_004319985.1	BHEV01023553.1	1260-1367	chr1	rep	Draupnirviridae_Krikovirus	Draupnirviridae-Krikovirus.001	Serpentes
Emydocephalus ijimae	GCA_004319985.1	BHEV01023553.1	1184-1237	chr1	rep	Draupnirviridae_Krikovirus	Draupnirviridae-Krikovirus.001	Serpentes
Hydrophis curtus	GCA_019472885.1	CM033602.1	88343903-88344043	chr1	rep	Draupnirviridae_Krikovirus	Draupnirviridae-Krikovirus.001	Serpentes
Hydrophis cyanocinctus	GCA_019473425.1	CM033584.1	84288055-84288195	chr1	rep	Draupnirviridae_Krikovirus	Draupnirviridae-Krikovirus.001	Serpentes
Laticauda colubrina	GCA_004320045.1	BHFR01000113.1	2975515-2975655	chr1	rep	Draupnirviridae_Krikovirus	Draupnirviridae-Krikovirus.001	Serpentes
Laticauda colubrina	GCA_015471245.1	BLBF01000005.1	53354276-53354416	chr1	rep	Draupnirviridae_Krikovirus	Draupnirviridae-Krikovirus.001	Serpentes
Laticauda laticaudata	GCA_004320025.1	BHFT01002131.1	598-738	chr1	rep	Draupnirviridae_Krikovirus	Draupnirviridae-Krikovirus.001	Serpentes
Naja naja	GCA_009733165.1	CM019148.1	297179533-297179673	chr1	rep	Draupnirviridae_Krikovirus	Draupnirviridae-Krikovirus.001	Serpentes
Naja naja	GCA_018093	JACCQE010000	7431928	chr1	rep	Draupnirviridae_Krikovirus	Draupnirviridae-	Serpentes

	825.1	001.1	1-74319421				Krikovirus.001	
Notechis scutatus	GCF_900518725.1	NW_020716647.1	4950572-4950712	chr1	rep	Draupnirviridae_Krikovirus	Draupnirviridae-Krikovirus.001	Serpentes
Notechis scutatus	GCA_900518725.1	ULFQ01000037.1	4950572-4950712	chr1	rep	Draupnirviridae_Krikovirus	Draupnirviridae-Krikovirus.001	Serpentes
Notechis scutatus	GCA_900608555.1	UWZA01000036.1	4962554-4962694	chr1	rep	Draupnirviridae_Krikovirus	Draupnirviridae-Krikovirus.001	Serpentes
Ophiophagus hannah	GCA_000516915.1	AZIM01001493.1	86626-86766	chr1	rep	Draupnirviridae_Krikovirus	Draupnirviridae-Krikovirus.001	Serpentes
Pantherophis guttatus	GCA_001185365.2	JTLQ02000034.1	35638987-35639172	chr1	rep	Draupnirviridae_Krikovirus	Draupnirviridae-Krikovirus.001	Serpentes
Pantherophis guttatus	GCF_001185365.1	NW_023010720.1	35638987-35639172	chr1	rep	Draupnirviridae_Krikovirus	Draupnirviridae-Krikovirus.001	Serpentes
Pantherophis obsoletus	GCA_012654085.1	WJSR01000066.1	35060735-35060920	chr1	rep	Draupnirviridae_Krikovirus	Draupnirviridae-Krikovirus.001	Serpentes
Pituophis catenifer	GCA_019677565.1	JAHUAB010000001.1	66369915-66370103	chr1	rep	Draupnirviridae_Krikovirus	Draupnirviridae-Krikovirus.001	Serpentes
Protobothrops flavoviridis	GCA_003402635.1	BFFQ01002846.1	28000-28188	chr1	rep	Draupnirviridae_Krikovirus	Draupnirviridae-Krikovirus.001	Serpentes
Protobothrops mucrosquamatus	GCA_001527695.3	LD637144.1	1386715-1386900	chr1	rep	Draupnirviridae_Krikovirus	Draupnirviridae-Krikovirus.001	Serpentes
Protobothro	GCF_001527	NW_015386571.	1386715-	chr1	rep	Draupnirviridae_Krikovirus	Draupnirviridae-	Serpentes

ps mucrosquamatus	695.2	1	1386900				Krikovirus.001	
Psammodynastes pulverulentus	GCA_025802295.1	JAOYMU010000001.1	96624646-96624827	chr1	rep	Draupnirviridae_Krikovirus	Draupnirviridae-Krikovirus.001	Serpentes
Pseudonaja textilis	GCF_900518735.1	NW_020769309.1	23164170-23164310	chr1	rep	Draupnirviridae_Krikovirus	Draupnirviridae-Krikovirus.001	Serpentes
Pseudonaja textilis	GCA_900518735.1	ULFR01000002.1	23164170-23164310	chr1	rep	Draupnirviridae_Krikovirus	Draupnirviridae-Krikovirus.001	Serpentes
Pseudonaja textilis	GCA_900608585.1	UWYZ01000002.1	23182362-23182502	chr1	rep	Draupnirviridae_Krikovirus	Draupnirviridae-Krikovirus.001	Serpentes
Ptyas mucosa	GCA_012654045.1	WNWU01000001.1	35068215-35068400	chr1	rep	Draupnirviridae_Krikovirus	Draupnirviridae-Krikovirus.001	Serpentes
Python bivittatus	GCF_000186305.1	NW_006537177.1	39161-39355	chr1	rep	Draupnirviridae_Krikovirus	Draupnirviridae-Krikovirus.001	Serpentes
Python bivittatus	GCA_000186305.2	KE958134.1	39161-39355	chr1	rep	Draupnirviridae_Krikovirus	Draupnirviridae-Krikovirus.001	Serpentes
Simalia boeleni	GCA_026541955.1	JANKYG010010711.1	33357-33707	chr1	rep	Draupnirviridae_Krikovirus	Draupnirviridae-Krikovirus.001	Serpentes
Thamnophis elegans	GCA_009769535.1	CM020096.1	110888776-110888966	chr1	rep	Draupnirviridae_Krikovirus	Draupnirviridae-Krikovirus.001	Serpentes
Thamnophis elegans	GCF_009769535.1	NC_045541.1	110888776-	chr1	rep	Draupnirviridae_Krikovirus	Draupnirviridae-Krikovirus.001	Serpentes

			1108889<br>66					
Thamnophis elegans	GCA_009769 695.1	WNNB0100601 1.1	794717-<br>794907	chr1	rep	Draupnirviridae_Krikovirus	Draupnirviridae-Krikovirus.001	Serpentes
Thamnophis sirtalis	GCA_001077 635.2	LFLD01S00119 7.1	239193-<br>239387	chr1	rep	Draupnirviridae_Krikovirus	Draupnirviridae-Krikovirus.001	Serpentes
Thamnophis sirtalis	GCF_001077 635.1	NW_013658876. 1	239193-<br>239387	chr1	rep	Draupnirviridae_Krikovirus	Draupnirviridae-Krikovirus.001	Serpentes
Thermophis baileyi	GCA_003457 575.1	QLTV01001682. 1	1797080-<br>1797268	chr1	rep	Draupnirviridae_Krikovirus	Draupnirviridae-Krikovirus.001	Serpentes
Vipera berus	GCA_000800 605.1	KN616336.1	72860-<br>73048	chr1	rep	Draupnirviridae_Krikovirus	Draupnirviridae-Krikovirus.001	Serpentes
Vipera latastei	GCA_024294 605.1	JANCQZ010002 442.1	148364-<br>148552	chr1	rep	Draupnirviridae_Krikovirus	Draupnirviridae-Krikovirus.001	Serpentes
Vipera latastei	GCA_024294 585.1	CM044354.1	7659976<br>5-<br>7659995<br>0	chr1	rep	Draupnirviridae_Krikovirus	Draupnirviridae-Krikovirus.001	Serpentes
Vipera ursinii	GCA_947247 035.1	OX365964.1	7332610<br>7-<br>7332629<br>5	chr1	rep	Draupnirviridae_Krikovirus	Draupnirviridae-Krikovirus.001	Serpentes
Elgaria multicarinat a	GCA_023053 635.1	JALIGS0100000 05.1	6828712<br>3-<br>6828744<br>3	chr5	cap	Draupnirviridae_Krikovirus_t ype_1a_cap	Draupnirviridae-Krikovirus.002	Anguimorpha
Elgaria multicarinat a	GCA_023053 675.1	JALIGT0100000 07.1	6747351<br>7-<br>6747383<br>7	chr5	cap	Draupnirviridae_Krikovirus_t ype_1a_cap	Draupnirviridae-Krikovirus.002	Anguimorpha
Heloderma charlesboge rti	GCA_026122 225.1	JANEZZ010000 438.1	439360-<br>439813	chr5	rep	Draupnirviridae_Krikovirus	Draupnirviridae-Krikovirus.002	Anguimorpha
Heloderma charlesboge	GCA_026122 225.1	JANEZZ010000 438.1	440333-<br>440608	chr5	cap	Draupnirviridae_Krikovirus_t ype_1a_cap	Draupnirviridae-Krikovirus.002	Anguimorpha

rti								
Shinisaurus crocodilurus	GCA_021292165.1	CM037880.1	173463852-173464339	chr5	rep	Draupnirviridae_Krikovirus	Draupnirviridae-Krikovirus.002	Anguimorpha
Shinisaurus crocodilurus	GCA_021292165.1	CM037880.1	173464466-173464636	chr5	cap	Draupnirviridae_Krikovirus_type_1a_cap	Draupnirviridae-Krikovirus.002	Anguimorpha
Heloderma charlesbogerti	GCA_026122225.1	JANEZZ010002294.1	1845167-1846095	chr2	rep	Draupnirviridae_Krikovirus	Draupnirviridae-Krikovirus.003	Anguimorpha
Heloderma charlesbogerti	GCA_026122225.1	JANEZZ010002294.1	1846354-1846871	chr2	cap	Draupnirviridae_Krikovirus_type_1a_cap	Draupnirviridae-Krikovirus.003	Anguimorpha
Shinisaurus crocodilurus	GCA_021292165.1	CM037877.1	151203773-151204590	chr2	rep	Draupnirviridae_Krikovirus	Draupnirviridae-Krikovirus.003	Anguimorpha
Shinisaurus crocodilurus	GCA_021292165.1	CM037877.1	151203291-151203596	chr2	cap	Draupnirviridae_Krikovirus_type_1a_cap	Draupnirviridae-Krikovirus.003	Anguimorpha
Heloderma charlesbogerti	GCA_026122225.1	JANEZZ010000138.1	6413-6574	chr3	rep	Draupnirviridae_Krikovirus	Draupnirviridae-Krikovirus.004	Anguimorpha
Shinisaurus crocodilurus	GCA_021292165.1	CM037878.1	92048988-92049772	chr3	rep	Draupnirviridae_Krikovirus	Draupnirviridae-Krikovirus.004	Anguimorpha
Elgaria multicarinata	GCA_023053635.1	JALIGS010000001.1	79084381-79084595	chr6	rep	Draupnirviridae_Krikovirus	Draupnirviridae-Krikovirus.005	Anguimorpha

Elgaria multicarinata	GCA_023053675.1	JALIGT010000002.1	79031948-79032162	chr6	rep	Draupnirviridae_Krikovirus	Draupnirviridae-Krikovirus.005	Anguimorpha
Elgaria multicarinata	GCA_023053635.1	JALIGS010000001.1	79084106-79084348	chr6	rep	Draupnirviridae_Krikovirus	Draupnirviridae-Krikovirus.005	Anguimorpha
Elgaria multicarinata	GCA_023053675.1	JALIGT010000002.1	79031673-79031915	chr6	rep	Draupnirviridae_Krikovirus	Draupnirviridae-Krikovirus.005	Anguimorpha
Heloderma charlesbogerti	GCA_026122225.1	JANEZZ010000462.1	1965116-1965804	chr6	rep	Draupnirviridae_Krikovirus	Draupnirviridae-Krikovirus.005	Anguimorpha
Heloderma charlesbogerti	GCA_026122225.1	JANEZZ010000462.1	1966334-1966600	chr6	cap	Draupnirviridae_Krikovirus_type_1a_cap	Draupnirviridae-Krikovirus.005	Anguimorpha
Shinisaurus crocodilurus	GCA_021292165.1	CM037881.1	96844550-96844750	chr6	cap	Draupnirviridae_Krikovirus_type_1a_cap	Draupnirviridae-Krikovirus.005	Anguimorpha
Pelusios castaneus	GCA_007922175.1	ML685866.1	234354-235213	chrUnk	rep	Draupnirviridae_Krikovirus	Draupnirviridae-Krikovirus.006	Pleurodira
Podocnemis expansa	GCA_007922195.1	ML682037.1	4546542-4547018	chrUnk	rep	Draupnirviridae_Krikovirus	Draupnirviridae-Krikovirus.006	Pleurodira
Podocnemis expansa	GCA_007922195.1	ML682037.1	4545693-4545827	chrUnk	rep	Draupnirviridae_Krikovirus	Draupnirviridae-Krikovirus.006	Pleurodira
Podocnemis expansa	GCA_007922195.1	ML682037.1	4545833-4545952	chrUnk	rep	Draupnirviridae_Krikovirus	Draupnirviridae-Krikovirus.006	Pleurodira
Pelusios castaneus	GCA_007922175.1	ML685784.1	5811865-5812481	chrUnk	rep	Draupnirviridae_Krikovirus	Draupnirviridae-Krikovirus.007	Pleurodira
Pelusios castaneus	GCA_007922175.1	ML685784.1	5812504-5812572	chrUnk	rep	Draupnirviridae_Krikovirus	Draupnirviridae-Krikovirus.007	Pleurodira

Pelusios castaneus	GCA_007922175.1	ML685784.1	5811247-5811488	chrUnk	cap	Draupnirviridae_Krikovirus_type_1a_cap	Draupnirviridae-Krikovirus.007	Pleurodira
Pelusios castaneus	GCA_007922175.1	ML685784.1	5812607-5812738	chrUnk	cap	Draupnirviridae_Krikovirus_type_1a_cap	Draupnirviridae-Krikovirus.007	Pleurodira
Podocnemis expansa	GCA_007922195.1	ML681998.1	26225829-26226605	chrUnk	rep	Draupnirviridae_Krikovirus	Draupnirviridae-Krikovirus.007	Pleurodira
Podocnemis expansa	GCA_007922195.1	ML681998.1	26225225-26225461	chrUnk	cap	Draupnirviridae_Krikovirus_type_1a_cap	Draupnirviridae-Krikovirus.007	Pleurodira
Podocnemis expansa	GCA_007922195.1	ML681998.1	26226624-26226734	chrUnk	cap	Draupnirviridae_Krikovirus_type_1a_cap	Draupnirviridae-Krikovirus.007	Pleurodira
Shinisaurus crocodilurus	GCA_021292165.1	CM037882.1	57165325-57165960	chr7	rep	Draupnirviridae_Krikovirus	Draupnirviridae-Krikovirus.008	Shinisaurus_crocodilurus
Shinisaurus crocodilurus	GCA_021292165.1	CM037882.1	57165224-57165286	chr7	rep	Draupnirviridae_Krikovirus	Draupnirviridae-Krikovirus.008	Shinisaurus_crocodilurus
Charina bottae	GCA_023362775.1	JALMGJ010000003.1	111482434-111482820	chrUnk	rep	Draupnirviridae_Krikovirus	Draupnirviridae-Krikovirus.009	Henophidia
Charina bottae	GCA_023362785.1	JALMGK010000001.1	10928365-10928751	chrUnk	rep	Draupnirviridae_Krikovirus	Draupnirviridae-Krikovirus.009	Henophidia
Python bivittatus	GCA_000186305.2	KE955255.1	178788-178967	chrUnk	rep	Draupnirviridae_Krikovirus	Draupnirviridae-Krikovirus.009	Henophidia
Python	GCF_000186	NW_006534298.	178788-	chrUnk	rep	Draupnirviridae_Krikovirus	Draupnirviridae-	Henophidia

bivittatus	305.1	1	178967				Krikovirus.009	
Heloderma charlesbogerti	GCA_026122225.1	JANEZZ010003345.1	1082131-1082349	chrUnk	cap	Draupnirviridae_Krikovirus_type_1a_cap	Draupnirviridae-Krikovirus.010	Heloderma_charlesbogerti
Heloderma charlesbogerti	GCA_026122225.1	JANEZZ010003345.1	1078831-1078983	chrUnk	cap	Draupnirviridae_Krikovirus_type_1a_cap	Draupnirviridae-Krikovirus.010	Heloderma_charlesbogerti
Charina bottae	GCA_023362775.1	JALMGJ010000008.1	38729031-38729243	chrUnk	cap	Draupnirviridae_Krikovirus_type_1a_cap	Draupnirviridae-Krikovirus.011	Charina_bottae
Charina bottae	GCA_023362785.1	JALMGK010000009.1	38347409-38347621	chrUnk	cap	Draupnirviridae_Krikovirus_type_1a_cap	Draupnirviridae-Krikovirus.011	Charina_bottae
Pelusios castaneus	GCA_007922175.1	ML685767.1	27068787-27069334	chrUnk	cap	Draupnirviridae_Krikovirus_type_1a_cap	Draupnirviridae-Krikovirus.012	Pelusios_castaneus
Pelusios castaneus	GCA_007922175.1	ML685837.1	3997600-3998417	chrUnk	rep	Draupnirviridae_Krikovirus	Draupnirviridae-Krikovirus.013	Pelusios_castaneus
Pelusios castaneus	GCA_007922175.1	ML685837.1	3996490-3996809	chrUnk	cap	Draupnirviridae_Krikovirus_type_1a_cap	Draupnirviridae-Krikovirus.013	Pelusios_castaneus
Pelusios castaneus	GCA_007922175.1	ML685837.1	3998612-3998737	chrUnk	cap	Draupnirviridae_Krikovirus_type_1a_cap	Draupnirviridae-Krikovirus.013	Pelusios_castaneus
Forficula auricularia	GCA_024734495.1	JALMQS010000153.1	26445794-26445937	chrUnk	rep	Draupnirviridae_Krikovirus	Draupnirviridae-Krikovirus.014	Forficula_auricularia
Forficula auricularia	GCA_023213275.1	JAKCPH010000110.1	60298-60441	chrUnk	rep	Draupnirviridae_Krikovirus	Draupnirviridae-Krikovirus.014	Forficula_auricularia
Oryctes rhinoceros	GCA_019329825.1	JAHRIJ010000004.1	4775750-4775857	chrUnk	rep	Draupnirviridae_Krikovirus	Draupnirviridae-Krikovirus.015	Oryctes_rhinoceros
Oryctes	GCA_020654	JAIOKQ010001	5704547-	chrUnk	rep	Draupnirviridae_Krikovirus	Draupnirviridae-	Oryctes_rhinoceros

rhinoceros	165.1	012.1	5704654				Krikovirus.015	
Oryctes borbonicus	GCA_001443 705.1	LJIG01000821.1	43966-44530	chrUnk	rep	Draupnirviridae_Krikovirus	Draupnirviridae-Krikovirus.016	Oryctes_borbonicu s
Oryctes borbonicus	GCA_902654 985.2	LR736857.1	1639488 1-1639572 2	chrUnk	rep	Draupnirviridae_Krikovirus	Draupnirviridae-Krikovirus.016	Oryctes_borbonicu s
Oryctes borbonicus	GCA_001443 705.1	LJIG01000821.1	44617-44784	chrUnk	rep	Draupnirviridae_Krikovirus	Draupnirviridae-Krikovirus.016	Oryctes_borbonicu s
Oryctes borbonicus	GCA_001443 705.1	LJIG01003452.1	45487-46062	chrUnk	rep	Draupnirviridae_Krikovirus	Draupnirviridae-Krikovirus.017	Oryctes
Oryctes borbonicus	GCA_902654 985.2	LR736908.1	340706-341281	chrUnk	rep	Draupnirviridae_Krikovirus	Draupnirviridae-Krikovirus.017	Oryctes
Oryctes rhinoceros	GCA_019329 825.1	JAHRIJ0100000 52.1	285881-286446	chrUnk	rep	Draupnirviridae_Krikovirus	Draupnirviridae-Krikovirus.017	Oryctes
Oryctes rhinoceros	GCA_020654 165.1	JAIOKQ010001 013.1	5773088-5773653	chrUnk	rep	Draupnirviridae_Krikovirus	Draupnirviridae-Krikovirus.017	Oryctes
Oryctes rhinoceros	GCA_020654 165.1	JAIOKQ010000 691.1	476104-476982	chrUnk	rep	Draupnirviridae_Krikovirus	Draupnirviridae-Krikovirus.018	Oryctes_rhinoceros
Oryctes rhinoceros	GCA_019329 825.1	JAHRIJ0100000 03.1	7429581-7430485	chrUnk	rep	Draupnirviridae_Krikovirus	Draupnirviridae-Krikovirus.018	Oryctes_rhinoceros
Oryctes rhinoceros	GCA_019329 825.1	JAHRIJ0100000 04.1	2341142-2341919	chrUnk	rep	Draupnirviridae_Krikovirus	Draupnirviridae-Krikovirus.019	Oryctes_rhinoceros
Oryctes rhinoceros	GCA_019329 825.1	JAHRIJ0100000 04.1	2340860-2341117	chrUnk	cap	Draupnirviridae_Krikovirus_t ype_1b_cap	Draupnirviridae-Krikovirus.019	Oryctes_rhinoceros
Oryctes rhinoceros	GCA_020654 165.1	JAIOKQ010000 067.1	4549129-4549740	chrUnk	rep	Draupnirviridae_Krikovirus	Draupnirviridae-Krikovirus.020	Oryctes_rhinoceros
Oryctes rhinoceros	GCA_019329 825.1	JAHRIJ0100000 05.1	8115279-8115776	chrUnk	rep	Draupnirviridae_Krikovirus	Draupnirviridae-Krikovirus.020	Oryctes_rhinoceros
Oryctes rhinoceros	GCA_020654 165.1	JAIOKQ010000 053.1	3767738-3768322	chrUnk	rep	Draupnirviridae_Krikovirus	Draupnirviridae-Krikovirus.021	Oryctes_rhinoceros
Oryctes rhinoceros	GCA_020654 165.1	JAIOKQ010000 053.1	3768418-3768819	chrUnk	rep	Draupnirviridae_Krikovirus	Draupnirviridae-Krikovirus.021	Oryctes_rhinoceros
Oryctes	GCA_019329	JAHRIJ0100000	6795186-	chrUnk	rep	Draupnirviridae_Krikovirus	Draupnirviridae-	Oryctes_rhinoceros

rhinoceros	825.1	06.1	6795705				Krikovirus.021	
Oryctes rhinoceros	GCA_020654 165.1	JAIOKQ010000 004.1	828895- 829440	chrUnk	rep	Draupnirviridae_Krikovirus	Draupnirviridae- Krikovirus.022	Oryctes_rhinoceros
Oryctes rhinoceros	GCA_020654 165.1	JAIOKQ010000 004.1	827681- 828082	chrUnk	rep	Draupnirviridae_Krikovirus	Draupnirviridae- Krikovirus.022	Oryctes_rhinoceros
Oryctes rhinoceros	GCA_020654 165.1	JAIOKQ010000 004.1	829708- 829962	chrUnk	cap	Draupnirviridae_Krikovirus_t ype_1b_cap	Draupnirviridae- Krikovirus.022	Oryctes_rhinoceros
Marronus borbonicus	GCA_902655 005.2	LR737382.1	1841062 6- 1841081 1	chrUnk	rep	Draupnirviridae_Krikovirus	Draupnirviridae- Krikovirus.023	Dynastinae
Marronus borbonicus	GCA_902655 005.2	LR737382.1	1841000 7- 1841017 7	chrUnk	rep	Draupnirviridae_Krikovirus	Draupnirviridae- Krikovirus.023	Dynastinae
Marronus borbonicus	GCA_902655 005.2	LR737382.1	1841036 7- 1841055 5	chrUnk	rep	Draupnirviridae_Krikovirus	Draupnirviridae- Krikovirus.023	Dynastinae
Oryctes borbonicus	GCA_902654 985.2	LR736870.1	4560870- 4561283	chrUnk	rep	Draupnirviridae_Krikovirus	Draupnirviridae- Krikovirus.023	Dynastinae
Oryctes borbonicus	GCA_001443 705.1	LJIG01000918.1	5917- 6237	chrUnk	rep	Draupnirviridae_Krikovirus	Draupnirviridae- Krikovirus.023	Dynastinae
Oryctes borbonicus	GCA_001443 705.1	LJIG01000918.1	5594- 5869	chrUnk	rep	Draupnirviridae_Krikovirus	Draupnirviridae- Krikovirus.023	Dynastinae
Oryctes borbonicus	GCA_001443 705.1	LJIG01000918.1	6240- 6440	chrUnk	rep	Draupnirviridae_Krikovirus	Draupnirviridae- Krikovirus.023	Dynastinae
Marronus borbonicus	GCA_902655 005.2	LR737389.1	182347- 182844	chrUnk	cap	Draupnirviridae_Krikovirus_t ype_1b_cap	Draupnirviridae- Krikovirus.024	Dynastinae
Oryctes borbonicus	GCA_902654 985.2	LR736934.1	300157- 300654	chrUnk	cap	Draupnirviridae_Krikovirus_t ype_1b_cap	Draupnirviridae- Krikovirus.024	Dynastinae
Oryctes borbonicus	GCA_001443 705.1	LJIG01007520.1	2836- 3156	chrUnk	cap	Draupnirviridae_Krikovirus_t ype_1b_cap	Draupnirviridae- Krikovirus.024	Dynastinae
Oryctes	GCA_001443	LJIG01007520.1	2664-	chrUnk	cap	Draupnirviridae_Krikovirus_t	Draupnirviridae-	Dynastinae

borbonicus	705.1		2807			ype_1b_cap	Krikovirus.024	
Marronus borbonicus	GCA_902655 005.2	LR737387.1	1336585 8- 1336623 2	chrUnk	rep	Draupnirviridae_Krikovirus	Draupnirviridae-Krikovirus.025	Dynastinae
Oryctes borbonicus	GCA_902654 985.2	LR736862.1	1250163 5- 1250189 3	chrUnk	rep	Draupnirviridae_Krikovirus	Draupnirviridae-Krikovirus.025	Dynastinae
Oryctes borbonicus	GCA_001443 705.1	LJIG01012873.1	16192- 16390	chrUnk	rep	Draupnirviridae_Krikovirus	Draupnirviridae-Krikovirus.025	Dynastinae
Oryctes borbonicus	GCA_001443 705.1	LJIG01012873.1	16092- 16178	chrUnk	rep	Draupnirviridae_Krikovirus	Draupnirviridae-Krikovirus.025	Dynastinae
Marronus borbonicus	GCA_902655 005.2	LR737417.1	637709- 637993	chrUnk	rep	Draupnirviridae_Krikovirus	Draupnirviridae-Krikovirus.026	Dynastinae
Oryctes borbonicus	GCA_001443 705.1	LJIG01016062.1	64128- 64469	chrUnk	rep	Draupnirviridae_Krikovirus	Draupnirviridae-Krikovirus.026	Dynastinae
Oryctes borbonicus	GCA_902654 985.2	LR736909.1	1028004- 1028327	chrUnk	rep	Draupnirviridae_Krikovirus	Draupnirviridae-Krikovirus.026	Dynastinae
Oryctes borbonicus	GCA_001443 705.1	LJIG01024665.1	14967- 15500	chrUnk	cap	Draupnirviridae_Krikovirus_t ype_1b_cap	Draupnirviridae-Krikovirus.027	Oryctes_borbonicu s
Oryctes borbonicus	GCA_001443 705.1	LJIG01025879.1	765-992	chrUnk	cap	Draupnirviridae_Krikovirus_t ype_1b_cap	Draupnirviridae-Krikovirus.028	Oryctes_borbonicu s
Oryctes borbonicus	GCA_001443 705.1	LJIG01025879.1	688-744	chrUnk	cap	Draupnirviridae_Krikovirus_t ype_1b_cap	Draupnirviridae-Krikovirus.028	Oryctes_borbonicu s
Oryctes borbonicus	GCA_902654 985.2	LR736892.1	187493- 187867	chrUnk	rep	Draupnirviridae_Krikovirus	Draupnirviridae-Krikovirus.029	Oryctes_borbonicu s
Oryctes borbonicus	GCA_902654 985.2	LR736892.1	188241- 188483	chrUnk	cap	Draupnirviridae_Krikovirus_t ype_1b_cap	Draupnirviridae-Krikovirus.029	Oryctes_borbonicu s
Marronus borbonicus	GCA_902655 005.2	LR737432.1	416523- 416696	chrUnk	rep	Draupnirviridae_Krikovirus	Draupnirviridae-Krikovirus.030	Dynastinae
Marronus borbonicus	GCA_902655 005.2	LR737432.1	415995- 416477	chrUnk	cap	Draupnirviridae_Krikovirus_t ype_1b_cap	Draupnirviridae-Krikovirus.030	Dynastinae
Oryctes	GCA_902654	LR736940.1	310139-	chrUnk	cap	Draupnirviridae_Krikovirus_t	Draupnirviridae-	Dynastinae

borbonicus	985.2		310411			ype_1b_cap	Krikovirus.030	
Oryctes borbonicus	GCA_902654 985.2	LR736940.1	309946-310137	chrUnk	cap	Draupnirviridae_Krikovirus_t ype_1b_cap	Draupnirviridae-Krikovirus.030	Dynastinae
Marronus borbonicus	GCA_902655 005.2	LR737383.1	1228115 6-1228129 0	chrUnk	rep	Draupnirviridae_Krikovirus	Draupnirviridae-Krikovirus.031	Marronus_borbonic us
Marronus borbonicus	GCA_902655 005.2	LR737391.1	6063394-6063837	chrUnk	cap	Draupnirviridae_Krikovirus_t ype_1b_cap	Draupnirviridae-Krikovirus.032	Marronus_borbonic us
Marronus borbonicus	GCA_902655 005.2	LR737392.1	6036562-6037005	chrUnk	cap	Draupnirviridae_Krikovirus_t ype_1b_cap	Draupnirviridae-Krikovirus.032	Marronus_borbonic us
Marronus borbonicus	GCA_902655 005.2	LR737395.1	1713408-1714220	chrUnk	rep	Draupnirviridae_Krikovirus	Draupnirviridae-Krikovirus.033	Marronus_borbonic us
Marronus borbonicus	GCA_902655 005.2	LR737408.1	1222812-1223663	chrUnk	rep	Draupnirviridae_Krikovirus	Draupnirviridae-Krikovirus.034	Marronus_borbonic us
Marronus borbonicus	GCA_902655 005.2	LR737408.1	1224357-1224959	chrUnk	rep	Draupnirviridae_Krikovirus	Draupnirviridae-Krikovirus.034	Marronus_borbonic us
Melolontha melolontha	GCA_935421 215.2	OW285242.1	5225165 8-5225236 1	chr8	rep	Draupnirviridae_Krikovirus	Draupnirviridae-Krikovirus.035	Melolontha_melolo ntha
Melolontha melolontha	GCA_935421 215.2	OW285242.1	5226709 8-5226856 3	chr8	rep	Draupnirviridae_Krikovirus	Draupnirviridae-Krikovirus.036	Melolontha_melolo ntha
Melolontha melolontha	GCA_935421 215.2	OW285242.1	5226599 5-5226680 1	chr8	rep	Draupnirviridae_Krikovirus	Draupnirviridae-Krikovirus.036	Melolontha_melolo ntha
Melolontha melolontha	GCA_935421 215.2	OW285242.1	5228132 6-5228213 2	chr8	rep	Draupnirviridae_Krikovirus	Draupnirviridae-Krikovirus.037	Melolontha_melolo ntha
Melolontha	GCA_935421	OW285242.1	5228307	chr8	rep	Draupnirviridae_Krikovirus	Draupnirviridae-	Melolontha_melolo

melolontha	215.2		9-5228389 0				Krikovirus.037	ntha
Melolontha melolontha	GCA_935421 215.2	OW285242.1	5228242 9-5228303 7	chr8	cap	Draupnirviridae_Krikovirus_t ype_1b_cap	Draupnirviridae-Krikovirus.037	Melolontha_melolo ntha
Melolontha melolontha	GCA_935421 215.2	OW285242.1	5231015 0-5231098 4	chr8	rep	Draupnirviridae_Krikovirus	Draupnirviridae-Krikovirus.038	Melolontha_melolo ntha
Melolontha melolontha	GCA_935421 215.2	OW285242.1	5232711 2-5232813 0	chr8	rep	Draupnirviridae_Krikovirus	Draupnirviridae-Krikovirus.039	Melolontha_melolo ntha
Melolontha melolontha	GCA_935421 215.2	OW285242.1	5232648 4-5232705 6	chr8	rep	Draupnirviridae_Krikovirus	Draupnirviridae-Krikovirus.039	Melolontha_melolo ntha
Melolontha melolontha	GCA_935421 215.2	OW285242.1	5233745 4-5233773 5	chr8	cap	Draupnirviridae_Krikovirus_t ype_1b_cap	Draupnirviridae-Krikovirus.040	Melolontha_melolo ntha
Melolontha melolontha	GCA_935421 215.2	OW285242.1	5234090 7-5234170 3	chr8	rep	Draupnirviridae_Krikovirus	Draupnirviridae-Krikovirus.041	Melolontha_melolo ntha
Melolontha melolontha	GCA_935421 215.2	OW285242.1	5237864 4-5237935 1	chr8	rep	Draupnirviridae_Krikovirus	Draupnirviridae-Krikovirus.042	Melolontha_melolo ntha
Melolontha melolontha	GCA_935421 255.1	CAKXYX01000 0425.1	387504-388175	chr8	rep	Draupnirviridae_Krikovirus	Draupnirviridae-Krikovirus.043	Melolontha_melolo ntha
Podocnemis expansa	GCA_007922 195.1	ML681964.1	4491856 3-	chrUnk	rep	Draupnirviridae_Krikovirus	Draupnirviridae-Krikovirus.044	Podocnemis_expan sa

			44918913					
Polymyxa betae	GCA_003693705.1	RBZT01000154.1	28858-29408	chrUnk	cap	putative_Draupnirviridae_linked_Cap	putativeDraupnirviridae.997	Polymyxa_betae
Chromera velia	GCA_018398765.1	JAHBMJ010001660.1	125854-126522	chrUnk	cap	putative_Draupnirviridae_linked_Cap	putativeDraupnirviridae.998	Chromera_velia
Phrynocephalus versicolor	GCA_023846285.1	JAHTLZ010000240.1	2857558-2857629	chrUnk	rep	putative_Draupnirviridae_Krikovirus	putativeDraupnirviridae.999	Phrynocephalus_versicolor
